# Fecal Microbiota Transplantation in Neurological Disorders

**DOI:** 10.3389/fcimb.2020.00098

**Published:** 2020-03-24

**Authors:** Karuna E. W. Vendrik, Rogier E. Ooijevaar, Pieter R. C. de Jong, Jon D. Laman, Bob W. van Oosten, Jacobus J. van Hilten, Quinten R. Ducarmon, Josbert J. Keller, Eduard J. Kuijper, Maria Fiorella Contarino

**Affiliations:** ^1^Department of Medical Microbiology, Leiden University Medical Center, Leiden, Netherlands; ^2^Netherlands Donor Feces Bank, Leiden University Medical Center, Leiden, Netherlands; ^3^Centre for Infectious Disease Control, National Institute for Public Health and the Environment (Rijksinstituut voor Volksgezondheid en Milieu, RIVM), Bilthoven, Netherlands; ^4^Department of Gastroenterology, Amsterdam University Medical Centers, VU University Medical Center, Amsterdam, Netherlands; ^5^Department of Neurology, Leiden University Medical Center, Leiden, Netherlands; ^6^Department Biomedical Sciences of Cells & Systems, University Medical Center Groningen, Groningen, Netherlands; ^7^Department of Neurology, Amsterdam University Medical Centers, VU University Medical Center, Amsterdam, Netherlands; ^8^Center for Microbiome Analyses and Therapeutics, Leiden University Medical Center, Leiden, Netherlands; ^9^Department of Gastroenterology and Hepatology, Leiden University Medical Center, Leiden, Netherlands; ^10^Department of Gastroenterology, Haaglanden Medical Center, The Hague, Netherlands; ^11^Department of Neurology, Haga Teaching Hospital, The Hague, Netherlands

**Keywords:** fecal microbiota transplantation, nervous system diseases, gastrointestinal microbiome, neurodegenerative, autoimmunity, gut-brain axis, Parkinson's disease, autism spectrum disorder

## Abstract

**Background:** Several studies suggested an important role of the gut microbiota in the pathophysiology of neurological disorders, implying that alteration of the gut microbiota might serve as a treatment strategy. Fecal microbiota transplantation (FMT) is currently the most effective gut microbiota intervention and an accepted treatment for recurrent *Clostridioides difficile* infections. To evaluate indications of FMT for patients with neurological disorders, we summarized the available literature on FMT. In addition, we provide suggestions for future directions.

**Methods:** In July 2019, five main databases were searched for studies and case descriptions on FMT in neurological disorders in humans or animal models. In addition, the ClinicalTrials.gov website was consulted for registered planned and ongoing trials.

**Results:** Of 541 identified studies, 34 were included in the analysis. Clinical trials with FMT have been performed in patients with autism spectrum disorder and showed beneficial effects on neurological symptoms. For multiple sclerosis and Parkinson's disease, several animal studies suggested a positive effect of FMT, supported by some human case reports. For epilepsy, Tourette syndrome, and diabetic neuropathy some studies suggested a beneficial effect of FMT, but evidence was restricted to case reports and limited numbers of animal studies. For stroke, Alzheimer's disease and Guillain-Barré syndrome only studies with animal models were identified. These studies suggested a potential beneficial effect of healthy donor FMT. In contrast, one study with an animal model for stroke showed increased mortality after FMT. For Guillain-Barré only one study was identified. Whether positive findings from animal studies can be confirmed in the treatment of human diseases awaits to be seen. Several trials with FMT as treatment for the above mentioned neurological disorders are planned or ongoing, as well as for amyotrophic lateral sclerosis.

**Conclusions:** Preliminary literature suggests that FMT may be a promising treatment option for several neurological disorders. However, available evidence is still scanty and some contrasting results were observed. A limited number of studies in humans have been performed or are ongoing, while for some disorders only animal experiments have been conducted. Large double-blinded randomized controlled trials are needed to further elucidate the effect of FMT in neurological disorders.

## Introduction

The bidirectional communication between the gut and the central nervous system, often referred to as the gut-brain axis, has been a topic of great interest in the past decade. Several studies suggest an important role of the gut microbiota in the pathophysiology of neurological disorders. A different human gut microbiota composition compared to healthy controls has been reported for several neurological disorders, such as Parkinson's disease (Hasegawa et al., 2015; Keshavarzian et al., 2015; Scheperjans et al., 2015; Unger et al., 2016), multiple sclerosis (Miyake et al., 2015; Chen et al., 2016; Jangi et al., 2016; Cosorich et al., 2017), autism spectrum disorder (Finegold et al., 2002, 2010; De Angelis et al., 2013, 2015; Kang et al., 2013; Ma et al., 2019), Alzheimer's disease (Vogt et al., 2017; Zhuang et al., 2018; Haran et al., 2019; Li B. et al., 2019; Liu et al., 2019), neuromyelitis optica (Cree et al., 2016), Rett syndrome (Strati et al., 2016), epilepsy (Xie et al., 2017; Peng et al., 2018; Lindefeldt et al., 2019), amyotrophic lateral sclerosis (Fang et al., 2016; Rowin et al., 2017; Mazzini et al., 2018), cerebral infarction (Karlsson et al., 2012; Yin et al., 2015), spinal cord injury (Gungor et al., 2016), and multiple system atrophy (Tan et al., 2018). However, data on microbiota composition are frequently inconsistent and numerous potential confounders are involved. Interestingly, patients with these neurological disorders often experience gastrointestinal symptoms (Poewe, 2008; Adams et al., 2011; Postuma et al., 2012; McElhanon et al., 2014; Willison et al., 2016), which could imply that the intestinal tract is involved in disease pathophysiology. Onset, clinical characteristics and progression of these neurological disorders may potentially be modulated by gut microbiota interventions. Gut microbiota interventions could also affect availability and pharmacokinetics of medication for neurological disorders, which may lead to an increased efficacy and a different side effect profile. There are multiple gut microbiota interventions, e.g., the administration of antibiotics, probiotics, prebiotics, synbiotics, or fecal microbiota transplantation (FMT). Antibiotic treatment has been reported to change disease course in a few neurological disorders (Sandler et al., 2000; Laake and Oeksengaard, 2002; Fasano et al., 2013; Ghanizadeh and Berk, 2015; Angelucci et al., 2019; Lum et al., 2019). Probiotics may improve disease symptoms, but results are inconsistent (Parracho et al., 2010; Kaluzna-Czaplinska and Blaszczyk, 2012; West et al., 2013; Partty et al., 2015; Jiang et al., 2017; Shaaban et al., 2018; Gazerani, 2019; Kobayashi et al., 2019a,b; Tamtaji et al., 2019). The most effective option in modulation of the gut microbiota is FMT, which includes administration of a solution of fecal matter from a donor into the intestinal tract of a recipient. FMT is an efficacious treatment for recurrent *Clostridioides difficile* infections (van Nood et al., 2013; Kelly et al., 2016). It is currently under investigation for several neurological disorders. Publications on FMT in humans with and animal models of neurological disorders are discussed in this narrative review.

## Methods

### Data Sources and Search Strategy

In July 2019, a literature search on FMT in neurological disorders was performed in five main databases, including Pubmed, Embase, Web of Science, COCHRANE library and Academic Search Premier database, using appropriate keywords (Appendix 1). Meeting and congress abstracts were also included. Furthermore, the reference lists of some recent reviews were consulted to detect relevant additional publications. In addition, the website ClinicalTrials.gov was searched (June 27th 2019) for ongoing or planned clinical trials with FMT in neurological disorders. Further details of the search strategy are provided in Appendix 1.

### Study Selection

Eligibility was assessed by screening titles and abstracts. The following inclusion criteria were applied: (1) *in vivo* studies or case descriptions with FMT in humans or animal models; (2) FMT with feces from healthy humans or animals transferred to humans with, or animal models of, individual neurological disorders; or FMT with feces from humans with, or animal models of, individual neurological disorders transferred to healthy humans or animals; (3) original research; (4) articles in English.

Two exclusion criteria were applied: (1) Use of individual bacteria, bacterial groups or bacterial metabolites instead of feces; (2) the effect of FMT on neurological symptoms/features was not described.

## Results

### Search Results

**Figure 1:**
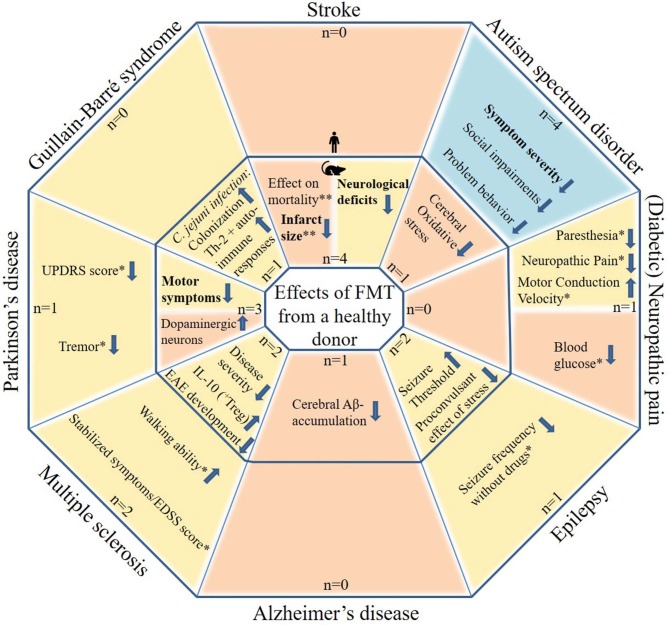
Potential effects of FMT in patients with neurological disorders and in animal models for neurological disorders. The figure includes studies in which patients with a neurological disorder or animal models for a neurological disorder received FMT with feces from a healthy donor. Tourette syndrome was not included as this contains only one case report. Blue areas include cognitive symptoms, yellow areas include motor and sensory symptoms or effects and orange areas include other effects. The outer parts contain results from human studies and the inner parts from animal studies. Statements in bold are found by more than one study, excluding case descriptions. N: the number of studies identified per neurological disorder, subdivided in human and animal studies. *based on case reports/series only (very limited evidence). **inconsistent results.

The initial search yielded 541 articles and abstracts. After exclusion of articles or abstracts not meeting the abovementioned criteria, 34 articles and abstracts remained. All included FMT studies are reported in Tables 1–**9**. Figure 1 shows the most important effects of FMT in humans and animals for neurological disorders. An overview of planned and ongoing studies, found on the website ClinicalTrials.gov, is provided in Appendix 2. Abbreviations and terms are explained in Appendix 3.

**Table 1:** FMT in autism spectrum disorder.

**Study design**	***N***	**Follow-up after FMT**	**Neurological effects of FMT**	**GI effects of FMT**	**FMT-effects on microbiota**	**SAE after FMT (animals: other important effects)**	**Pre-treatment**	**Administration route**	**No. of FMT**	**Amount of feces**	**Rationally selected feces donor**	**References**
Humans Open-label clinical trial. Relevant groups: FMT: 1) ASD children with moderate to severe GI symptoms No FMT: 2) Age- and gender-matched normally developing children without GI symptoms	38: 18 ASD (12 oral + 6 rectal route) and 20 controls	115 d Long-term: 2 y after completion of treatment	CARS, PGI-III, ABC, SRS, VABS-II: improved. No difference between oral or rectal delivery. Long-term: CARS, PGI-III, ABC, SRS, VABS-II: still improved compared to baseline and some compared to end of treatment.	GSRS: 77% reduction, DSR: 30% reduction in No. of days with abnormal feces. No difference between oral or rectal delivery. Long-term: GSRS: 58% reduction, DSR: 26% reduction.	α-diversity: FPD + observed OTUs increased, toward group 2. β-diversity: changed toward microbiota composition of donor (unw. UniFrac, not for w. UniFrac). Change in individual taxa: yes. Long-term:α-diversity: FPD + observed OTUs still increased. β-diversity: difference with donor similar to pre-FMT (Unw. UniFrac). Change in individual taxa: yes.	Temporary AE: Vancomycin: 39% mild to moderate hyperactivity, 28% mild to moderate tantrums/ aggression, 5% rash.Oral SHGM: 5% nausea. Long-term: NA.	AB: vancomycin Bowel lavage: yes	Upper GI route by mixing SHGM with a drink, lower GI route via enema.	7–8 w treatment	SHGM: Oral: 2.5 × 10^12^ cells/d for 2 d, then 2.5 × 10^9^ cells/d for 8 w. Rectal: single rectal dose of 2.5 × 10^12^ cells, after 1 w, 2.5 × 10^9^ cells/d orally for 7 w.	No	Kang et al., 2017, 2019
Humans Open-label, waitlist-controlled RCT (abstract only). Relevant groups: FMT: 1) ASD with FMT No FMT: 2) ASD with rehabilitation training 3) HC	62: 24 in group 1 and 2, 14 in group 3	4 m	CARS: 10.8% decrease in group 1, 0.8% decrease in group 2, remained marginally reduced after 2nd FMT (*P*= 0.074).	GSI: differences after 2 m	α-diversity: NA. β-diversity: NA. Change in individual taxa: yes, change toward group 3.	7 (29.2%) patients in group 1 with AE (fever, allergy, nausea), all mild and transient.	AB: NA Bowel lavage: NA	colonoscopy and gastroscopy under anesthesia	2	NA	No	Zhao et al., 2019
Humans Case series (abstract only)	9	Unclear	ASD symptoms: unchanged in 21 y.o., improved in one of two 8 y.o., improved in younger subjects on more long-lasting basis. Frequent regression, mostly after AB post-FMT, often improved after re-FMT.	NA	α-diversity: Shannon diversity slightly increased post-FMT (temporarily decreased after AB). β-diversity: not changed (except after AB). Change in individual taxa: yes.	No adverse effects	AB: vancomycin or vancomycin nitazoxanide colistin Bowel lavage: yes	Capsules and enema	First 12–24 capsules, 4 h later 1 enema, next d again capsule treatment	Capsules: 0.47 mL (>6 mL in total), Enema: 300–500 mL (50–100 mg feces).	No	Ward et al., 2016
Humans Case series (abstract only).	5	NA	PGI-R: referred as improved in all patients post-FMT (N.B.: no pre-FMT scores shown).	GSRS: improved in all patients post-FMT.	NA	None	AB: NA Bowel lavage: NA	Infusion into cecum (colonoscopy)	3	NA	No	Urbonas and Cervinskiene, 2018
Animal model: Offspring of mice with FMT from human ASD patients Relevant groups: (all GF WT mice) FMT: 1) Offspring human mild ASD-FMT 2) Offspring human ASD-FMT 3) Offspring human ND-FMT	14–121 per group per analysis	FMT at weaning, breeding at 7–8 w of age. Offspring were followed until P45.	MB, OFT, and USV: group 2 vs. other groups: more ASD-like behavioral deficits. 3-CST: No differences. DSI: decreased in group 2 vs. 3. Alternative splicing pattern of ASD-relevant genes in brainsof group 2 vs. 3.	Effects on GI symptoms NA. No differences in intestinal barrier function or cytokines from ileum or colon between group 2 and group 3.	Group 2 vs. 3: α-diversity: decreased (FPD and Pielou's evenness). β-diversity: different (unw. UniFrac+ Bray-Curtis). Difference in individual taxa: yes. Slight shift in α- and β-diversity in offspring vs. recipients.	NA	AB: NA Bowel lavage: NA	Oral gavage	1	100 μL per mouse	Feces from 8 human ASD children, 3 human mild ASD children or 5 human ND children.	Sharon et al., 2019
Animal model: PPA hamster model Relevant groups (all hamsters): FMT: 1) PPA+N-FMT No FMT: 2) Control 3) PPA 4) Clindamycin 5) PPA+bee pollen 6) PPA+Propolis, 7) PPA+ *L. paracaseii* 8) PPA+protexin	10 per group	4 w	More oxidative stress in brains of group 3 and 4 vs. all other groups.	NA	α-diversity: in group 1 increase in number of gut microbes at d 0 vs. group 2, but this decreased later. β-diversity: NA. Difference in individual taxa: yes.	NA	AB: NA Bowel lavage: NA	Anorectally	5	1 g in 10 mL PBS.	Feces from a normal hamster	Aabed et al., 2019

### Neurological Disorders With FMT Studies in Both Patients and Animal Models

#### Autism Spectrum Disorder

##### Role of the gut microbiota in disease symptoms and pathogenesis

Autism spectrum disorder (ASD) is a group of neurodevelopmental disorders, characterized by altered social communication and interaction as well as repetitive, stereotyped behavior.

The exact etiology is unknown: a combination of genetic and environmental risk factors, dysregulation of the immune system, inflammation and also maternal factors is proposed (Fattorusso et al., 2019). Increased systemic (Ashwood et al., 2011) and neuroinflammation (Vargas et al., 2005; Li et al., 2009) and even brain-specific autoantibodies (Vojdani et al., 2002; Silva et al., 2004; Connolly et al., 2006; Cabanlit et al., 2007; Wills et al., 2009), though not confirmed in another study (Todd et al., 1988), have been observed in ASD patients. Hyperserotoninemia in ASD patients may also contribute to the etiology (Fattorusso et al., 2019).

ASD patients have a different gut microbiota composition and different gut metabolomes (including neurotransmitters) compared to healthy controls (Finegold et al., 2002, 2010; De Angelis et al., 2013, 2015; Kang et al., 2013; Ma et al., 2019). Relatively higher levels of the phylum Bacteroidetes, which produces short-chain fatty acids (SCFA), are observed in ASD subjects. Furthermore, decreased levels of the anti-inflammatory genus *Bifidobacterium* or increased levels of the genus *Clostridium*, which is known to produce potentially toxic metabolites such as phenols and p-cresols, may play a role in pathogenesis (Fattorusso et al., 2019). Altered production of gut-microbial metabolites, such as p-cresol and SCFA, are associated with ASD symptoms (MacFabe et al., 2007; Fattorusso et al., 2019). An increased intestinal production of serotonin and decreased cerebral serotonin synthesis in ASD may also be caused by alterations in the gut microbiota, but evidence is inconsistent (Fattorusso et al., 2019). Data on α-diversity in gut microbiota of ASD patients are also contrasting (Finegold et al., 2010; Williams et al., 2011; De Angelis et al., 2013; Kang et al., 2013; Ma et al., 2019). An altered gut microbiota composition may influence the immune system, inflammation and metabolism and thereby increase the risk for ASD (Park, 2003; Fattorusso et al., 2019). Diet, which is known to shape the gut microbiota (David et al., 2014), is also thought to modulate ASD behavior (Knivsberg et al., 2002; Cermak et al., 2010; Whiteley et al., 2010; de Theije et al., 2014; Fattorusso et al., 2019).

Gastrointestinal symptoms, such as abdominal pain, constipation, diarrhea, and bloating, are more frequently described in ASD patients than controls, with a corresponding odds ratio of 4.42 (McElhanon et al., 2014). Some studies found an association between gastrointestinal symptoms and severity of ASD symptoms (Adams et al., 2011; Wang L. W. et al., 2011; Mazurek et al., 2013), but others could not reproduce these findings (Kang et al., 2013; Son et al., 2015). ASD patients appear to have a higher prevalence of IBD (Doshi-Velez et al., 2015) and a higher number of pro-inflammatory immune cells in the intestinal wall (Navarro et al., 2016), although intestinal inflammatory markers, such as fecal calprotectin, appear normal (Navarro et al., 2016). The gastrointestinal symptoms may be caused by the presence of more pro-inflammatory gut bacteria (Fattorusso et al., 2019), but other factors may also be involved (Mayer et al., 2014). Studies have also reported altered gastrointestinal motility and increased intestinal permeability in ASD (D'Eufemia et al., 1996; Boukthir et al., 2010; de Magistris et al., 2010), which may increase the risk of translocation of bacteria or neurotoxic peptides, such as lipopolysaccharide (LPS). However, inconsistency is again observed (Navarro et al., 2016).

Another important finding that supports a role for the gut microbiota is a temporary improvement of ASD and gastrointestinal symptoms after 8 weeks of oral vancomycin treatment (Sandler et al., 2000). Furthermore, several studies on the effect of probiotics in ASD patients and ASD animal models showed a positive effect on ASD symptoms (Parracho et al., 2010; Kaluzna-Czaplinska and Blaszczyk, 2012; Hsiao et al., 2013; West et al., 2013; Partty et al., 2015; Shaaban et al., 2018). This included improvement of neurobehavioral symptoms, such as anxiety or problems with concentration, and/or gastrointestinal symptoms (Parracho et al., 2010; Kaluzna-Czaplinska and Blaszczyk, 2012; Hsiao et al., 2013; West et al., 2013; Partty et al., 2015; Shaaban et al., 2018). One human study reported the absence of onset of Asperger syndrome in a group of 40 children of which the mothers during pregnancy or post-partum and, in case there was no breastfeeding, the children themselves had received probiotics (*Lactobacillus rhamnosus* GG) for 6 months as opposed to 3 out of 35 children who developed Asperger syndrome in the placebo group (Partty et al., 2015).

In animal studies, germ-free male mice showed increased social impairments compared to conventionally colonized mice, which suggests an important role for gut microbiota in this behavior (Desbonnet et al., 2014).

##### FMT studies in animal models (Table 1)

Sharon et al. (2019) performed FMT in germ-free wild-type mice with feces from children with ASD or normally developing children. The ASD group and their offspring had ASD-like symptoms. Furthermore, brains of offspring displayed alternative splicing of ASD-relevant genes. When gamma-aminobutyric acid (GABA)_A_ receptor agonists, reduced in the ASD-group colon, were administered to an ASD mouse model, ASD symptoms decreased. Another study (Aabed et al., 2019) observed decreased cerebral oxidative stress after FMT with feces from a normal hamster in an ASD hamster model. This effect was stronger after administration of *Lactobacillus paracaseii*.

##### FMT studies in patients (Table 1)

In an open-label clinical trial (Kang et al., 2017, 2019), 18 children with ASD and gastrointestinal symptoms received daily FMT for 7–8 weeks by mixing standardized human gut microbiota with a drink or via enema. Gastrointestinal and behavioral ASD symptoms improved, which persisted until 2 years after treatment. FMT appeared safe, since most adverse events were temporary and observed at start of vancomycin pre-treatment (e.g., mild to moderate tantrums/aggression and hyperactivity) and 5% suffered from nausea/vomiting. Furthermore, there was a correlation between ASD symptoms and gastrointestinal symptoms. However, this was an open-label study without a placebo group in a heterogenous group of 18 participants, in which 12 changed their medication, diet, or nutritional supplements during the study. Furthermore, there was no vancomycin-only group and no information on adverse events in the long-term follow-up was provided (Kang et al., 2017, 2019).

An abstract (Zhao et al., 2019) reporting an open-label, randomized waitlist-controlled trial showed improvements of ASD symptoms and changes in gastrointestinal symptoms 2 months after two FMTs in 24 ASD-children compared to 24 control ASD-children. However, improvement of ASD symptoms was temporary. Seven FMT-patients reported adverse events, such as nausea, fever and allergy, but these were all mild and transient. There was no placebo-group and there was lack of information on α- and β-diversity of the gut microbiota, pre-treatment and amount of donor feces (Zhao et al., 2019).

In a case series described in an abstract (Ward et al., 2016), ASD symptoms did not change in a 21-year-old man, but were improved in eight younger subjects. Regression of symptoms often occurred, mostly after antibiotics post-FMT, but often improved again after re-FMT. In another case series (Urbonas and Cervinskiene, 2018) (abstract only), the authors described that the parent global impression score (PGI-R) and gastrointestinal symptoms improved after three FMTs in five boys with ASD and mild gastrointestinal symptoms. However, PGI-R pre-FMT scores were not shown and scores did not appear to improve over time.

One placebo-controlled randomized clinical trial (RCT) with CP101, a drug that contains a full community of gut bacteria, is planned and two RCT, of which one is placebo-controlled, with FMT in human ASD subjects are ongoing (Appendix 2).

#### Multiple Sclerosis

##### Role of the gut microbiota in disease symptoms and pathogenesis

Multiple sclerosis (MS) is a demyelinating disorder of the central nervous system (CNS). The pathophysiology of MS is complex and has not been fully elucidated. Genetic, infectious and environmental factors (e.g., Epstein-Barr virus infection, smoking, and sunlight/vitamin D) play pivotal roles (Olsson et al., 2017). The interplay between these factors appears to lead to immune dysregulation, but a true autoimmune origin of the disease remains elusive. Nevertheless, the relative efficacy of therapies targeting inflammation supports a critical role of the immune system, in particular T-cell mediated mechanisms. This includes limiting leukocyte egress from secondary lymphoid organs and their entry into the CNS (Dendrou et al., 2015; Thompson et al., 2018). Peripheral activation of CD8^+^ T-cells and CD4^+^ T-helper cells (Th) type 1 and 17 allows for these cells to infiltrate the CNS and cause inflammation. This peripheral activation is thought to be caused by a reduction of regulatory T cells (Treg) and antigen presentation of brain antigens in secondary lymphoid organs (Dendrou et al., 2015). Infiltrating macrophages are also critical to CNS inflammation.

The gut microbiota influences and modulates the equilibrium between pro- and anti-inflammatory T-cells in the gut-associated lymphoid tissue (Rooks and Garrett, 2016; Yissachar et al., 2017). Several studies have been performed which characterized the gut microbiota profile of patients suffering from different forms of MS. Similar subtle alterations in gut microbiota composition were found throughout these studies via analyses of fecal samples (Miyake et al., 2015; Chen et al., 2016; Jangi et al., 2016). One study found a similar perturbed microbiota composition within duodenal mucosal biopsies (Cosorich et al., 2017). Based on these studies, it could be hypothesized that MS patients' gut microbiota harbors less bacterial species that can induce Treg cells, which may contribute to elevated peripheral levels of Th1 and 17 (Jangi et al., 2016; Cekanaviciute et al., 2017). It is hypothesized that subsequently elevated Th1 and 17 cause inflammation in the CNS and increased blood-brain barrier permeability, leading in turn to exacerbation of the inflammation of the CNS (Dendrou et al., 2015). Modulation of the gut microbiota to induce more Treg cells could lead to less activation of pathogenic T-cells (Berer et al., 2014). Interestingly, gavage with a human gut-derived commensal strain *Prevotella histicola* resulted in a decreased incidence of disease in a mouse model of MS (Mangalam et al., 2017). Moreover, a decrease in Th1 and 17 cell numbers and an increase in Treg cells were found.

##### FMT studies in animal models (Table 2)

Experimental autoimmune encephalomyelitis (EAE) is an animal model that mimics aspects of pathophysiology and symptoms of MS (Goverman et al., 1993). The gut microbiota is required to induce EAE, as germ-free mice did not develop spontaneous EAE in a transgenic model (Berer et al., 2011). Two mouse EAE studies used gavage to transplant MS patients' or healthy human controls' microbiota. The transplanted MS microbiota resulted in an increased EAE incidence, a more severe disease course and a decrease in expression of anti-inflammatory cytokine IL-10 (Berer et al., 2017; Cekanaviciute et al., 2017). In general, these findings seem to correlate with current interpretations of the distinct immunological findings in MS-patients (Dendrou et al., 2015).

**Table 2:** FMT in multiple sclerosis.

**Study design**	***N***	**Follow-up after FMT**	**Neurological effects of FMT**	**GI effects of FMT**	**FMT-effects on microbiota**	**SAE after FMT (animals: other important effects)**	**Pre-treatment**	**Administration route**	**No. of FMT**	**Amount of feces**	**Rationally selected feces donor**	**References**
Humans Case series (abstract only)	3	15 y, 3 y, 2 y	Improved walking ability in all cases, catheter not required anymore, increased energy levels. No relapses.	Constipation resolved	NA	None	AB: NA Bowel lavage: NA	NA	5, 10, 5	NA	NA	Borody et al., 2011
Human (SPMS) Case report	1	>10 y	EDSS score stabilized. Functional system scores and modified MSFC scores minimally improved.	NA	NA	None	AB: vancomycin Bowel lavage: no	Enema	1	NA	No	Makkawi et al., 2018
Animal model: Transgenic RR SJL/J mice carrying a MOG-specific T cell receptor Relevant groups: FMT (GF RR SJL/J mice): 1) MS-FMT 2) HT-FMT	38: 20 group 1, 18 group 2	12 w	Higher incidence of EAE onset in group 1. Increased IL-10 expression in group 2.	NA	α-diversity: less in groups 1 + 2 vs. donor, no comparison between group 1 and 2. β-diversity: clustering by donor and twin pair but not by EAE disease state (w. UniFrac+ PCoA). Difference in individual taxa: yes.	None	AB: no Bowel lavage: no	Oral gavage	1	1 g of feces, ≈300 μL of fecal suspension	5 pairs of MS-human donors+ healthy twin (MS-discordant monozygotic twins)	Berer et al., 2017
Animal model: Mice with EAE induction via immunization with MOG35-55 emulsion, mixed with CFA and killed mycobacterium tuberculosis H37Ra, followed by i.p injections of pertussis toxin. Relevant groups (all GF WT mice): FMT: 1) MS-FMT+MOG35-55 2) HHC-FMT+MOG35-55 No FMT: 3) MOG 35-55	6–8 per group	≈70 d (EAE induction at 35 d post-FMT)	More severe clinical course in group 1 vs. 2. Decrease in IL-10^+^ Treg in mesenteric lymph nodes in group 1 vs. 2.	NA	α-diversity: no difference in richness (Chao1). β-diversity: different between group 1 and 2 and donors (w. UniFrac+ PCoA). Difference in individual taxa: yes.	None	AB: ampicillin neomycin metronidazole vancomycin (ampho B) Bowel lavage: no	Gavage	1	NA	3 MS-human donors, and 3 human healthy household controls	Cekanaviciute et al., 2017

##### FMT studies in patients (Table 2)

Currently, there are only two case reports/series on effects of FMT on MS symptoms and disease progression (Borody et al., 2011; Makkawi et al., 2018). Both claim sustained beneficial effects following FMT. One secondary progressive MS patient was treated with a single FMT for concomitant recurrent *Clostridioides difficile* infections. The FMT resolved the recurrent *C. difficile* infections and was suggested to prevent MS disease progression for over 10 years (Makkawi et al., 2018). In the case-series amelioration of MS symptoms following repeated FMTs was observed in three patients (Borody et al., 2011).

ClinicalTrials.gov lists one ongoing RCT, one ongoing non-randomized trial, and one planned prospective case-only observational study in which MS patients receive FMT as an experimental treatment (Appendix 2).

#### Parkinson's Disease

##### Role of the gut microbiota in disease symptoms and pathogenesis

Parkinson's disease (PD) is a progressive neurodegenerative disorder, characterized by neuron degeneration in the CNS, enteric nervous system and peripheral autonomic nervous system, and by the presence of Lewy bodies and Lewy neurites in affected neurons (Pakkenberg et al., 1991). The etiology and pathogenesis of PD is still largely unknown and possibly heterogeneous. Both genetic and environmental factors might play a role, at least in some forms of the disease. The gut-brain axis in PD has been intensively studied. Gastrointestinal symptoms (including obstipation and delayed transit) are frequently observed in patients with PD. In some cases they precede the onset of motor symptoms and represent the first clinical manifestation of PD (Poewe, 2008; Postuma et al., 2012).

An important factor in the etiology of PD is the aggregation of the protein alpha-synuclein (αSyn), a major component of Lewy bodies (Spillantini et al., 1997). Several studies demonstrated that the enteric nervous system and vagus nerve are affected in an early, and even in the prodromal, phase of disease (Braak et al., 2003; Kuo et al., 2010; Hallett et al., 2012; Shannon et al., 2012; Stokholm et al., 2016). It has been suggested that the disease may start in the gut, with a neurotropic substance with prion-like properties, possibly misfolded αSyn, that is transported from the gastrointestinal tract to the CNS (Liautard, 1991; Braak et al., 2003). Mice studies indeed confirmed that αSyn forms could be transported to the brain and pass the blood-brain barrier (Pan-Montojo et al., 2010; Ulusoy et al., 2013; Holmqvist et al., 2014; Kim et al., 2019). Moreover, aggregation of αSyn in the brain, and possibly the gut, of PD patients could be a consequence of inflammation-induced oxidative stress (Shults, 2006; Keshavarzian et al., 2015). It is indeed observed that PD patients have increased expression of pro-inflammatory cytokines and glial markers in the colonic biopsies compared to healthy controls (Devos et al., 2013). PD patients also appear to have increased intestinal permeability (Forsyth et al., 2011) and small intestine bacterial overgrowth (Gabrielli et al., 2011; Fasano et al., 2013; Tan et al., 2014). The latter is associated with more motor fluctuations when using levodopa, which improves after antibiotics (Fasano et al., 2013). Furthermore, several studies indicate that the gut microbiota composition and the metabolome in PD patients are different from healthy individuals (Hasegawa et al., 2015; Keshavarzian et al., 2015; Scheperjans et al., 2015; Unger et al., 2016). Overall, more pro-inflammatory gut bacteria, such as LPS-producing Proteobacteria, and less anti-inflammatory butyrate-producing gut bacteria are found in PD patients (Keshavarzian et al., 2015). Scheperjans et al. (2015) found that the relative abundance of the family *Enterobacteriaceae* in PD patients is positively associated with postural instability and gait difficulty. Two other important studies suggested that gut bacterial tyrosine decarboxylases can metabolize levodopa to dopamine without being susceptible for aromatic amino acid decarboxylase inhibitors, such as carbidopa. Increased presence of gut bacterial tyrosine decarboxylases may thereby cause response fluctuations in levodopa/carbidopa-treated PD patients, as dopamine cannot cross the blood-brain barrier (Maini Rekdal et al., 2019; van Kessel et al., 2019). Furthermore, probiotics may improve PD symptoms, but this includes mainly improvement of constipation (Gazerani, 2019).

##### FMT studies in animal models (Table 3)

A recent study (Sampson et al., 2016) demonstrated that the presence of gut microbiota is necessary for the development of PD characteristics in alpha-synuclein-overexpressing (ASO) mice. Germ-free ASO mice showed less motor symptoms, constipation, alpha-synucleinopathy, and microglia activation compared to specific-pathogen-free ASO mice, while colonization with specific-pathogen-free microbiota led to an increase of symptoms. When ASO mice received feces from PD patients, motor symptoms increased compared to mice that received healthy human feces (Sampson et al., 2016). Another study (Sun et al., 2018) showed that a PD mouse model had improved motor function, increased striatal neurotransmitters, and decreased neuroinflammation after receiving feces from healthy mice. Healthy mice that received feces from PD mice had deteriorated motor function and decreased striatal neurotransmitters compared to controls. Zhou et al. (2019) observed less motor function decline and loss of dopaminergic neurons in the substantia nigra in PD mice that received a fasting mimicking diet (FMD) compared to *ad-libitum*-fed PD mice. Furthermore, they observed a higher striatal dopamine and serotonin concentration in PD mice that had received feces from FMD-fed control mice compared to phosphate-buffered solution (PBS)-gavaged or *ad-libitum* microbiota-gavaged PD mice.

**Table 3:** FMT in Parkinson's disease.

**Study design**	***N***	**Follow-up after FMT**	**Neurological effects of FMT**	**GI effects of FMT**	**FMT-effects on microbiota**	**SAE after FMT (animals: other important effects)**	**Pre-treatment**	**Administration route**	**No. of FMT**	**Amount of feces**	**Rationally selected feces donor**	**References**
Human Case report	1	3 m	UPDRS: decreased at 1 w after end of FMT-treatment, but became similar to pre-FMT at 3 m post-FMT. Leg tremor almost disappeared at 1 w post-FMT but recurred in right lower extremity, more mild than pre-FMT, at 2 m post-FMT.	Wexner constipation score: decreased from 16 to 10. PAC-QOL: decreased from 18 to 12 (8 at 1 w post-FMT). Defecation time: Decreased from >30 to 5 min.	α-diversity: increased 1 w post-FMT, decreased after 3 m (OTU Number). β-diversity: similar to donor at 1 w post-FMT, but similarity decreased later (w. UniFrac+PCoA). Difference in individual taxa: yes.	No adverse effects	AB: NA Bowel lavage: NA	TET tube, inserted into the ileocecal junction	3	200 mL	No	Huang H. et al., 2019
Animal model: Thy1-αSyn (ASO) mice Relevant groups: (all ASO or WT mice) FMT: 1) GF+SPF-WT-FMT 2) GF+human PD-FMT 3) GF+human HC-FMT No FMT: 4) GF 5) SPF 6) SPF+AB	3–12 per group per analysis	6–8 w (unclear for group 2 and 3)	Beam traversal, pole descent, adhesive removal, hindlimb clasping reflex score: ASO group 2 more motor symptoms vs. ASO group 3. No effects in WT mice. Beam traversal, pole descent, adhesive removal, hindlimb clasping reflex score: In ASO group 1 deterioration of motor symptoms and increased microglia cell body diameter, vs. WT group 1 and 4.	No difference in constipation between group 2 and 3 in ASO or WT mice.In ASO group 1 more constipation, vs. WT group 1 and 5 and WT or ASO group 4 and 6.	α-diversity: NA. β-diversity: most similar to donor, mice with PD donors more similar to each other than to mice with HC donors. Difference between ASO and WT-mice post-FMT (w. en unw. UniFrac+ Bray-Curtis). Difference in individual taxa: yes. FMT with feces from SPF WT mice: NA.	NA	AB: NA Bowel lavage: NA	Oral gavage	1	NA	Feces from 6 human PD patients, 6 human HCs or 3 SPF WT mice	Sampson et al., 2016
Animal model: MPTP-induced PD mice (i.p. injection) Relevant groups: (all SPF WT mice) FMT: 1) MPTP+HC-FMT 2) NS+PD-FMT 3) NS+HC-FMT No FMT: 4) No treatment 5) MPTP+PBS 6) NS+PBS	10–15 per group	8 d after first FMT (until 1st d after last treatment)	Worsened performance in pole descent and traction test and reduced striatal neurotransmitters in group 5 and 2 vs. group 4, 6 and 3. Also improved (including no of dopaminergic neurons) in group 1 vs. group 5. Neuroinflammation: decreased activated astrocytes and microglia in SN and reduced expression of TLR4/TNF-α signaling pathway components in gut and brain in group 1 vs. group 5.	NA	α-diversity: Trend to increase in group 4 and little increase in group 1 vs. 5 (Chao-1, phylog. div. whole tree). β-diversity: clustering of group 1, group 4 and group 5 (w. UniFrac+ PCoA). Difference in individual taxa: yes.	NA	AB: NA Bowel lavage: NA	Gavage	7	200 μL	Feces from normal control mice or MPTP-induced PD mice	Sun et al., 2018
Animal model: MPTP-induced PD mice (i.p. injection) Relevant groups: FMT (AB-treated WT mice): 1) MPTP+AL-FMT 2) MPTP+FMD-FMT No FMT (WT mice): 3) AB+MPTP+PBS/G 4) AB+MPTP+NF/HK	8 per group	8 d after first FMT (until 1st d after last treatment)	Striatal DA and 5-HT concentration of group 2 higher than group 1 and 3. 5-HT concentration increased in group 1 compared with group 3. 5-HT concentration decreased in group 4, compared with group 2.	NA	NA	NA	AB: bacitracin gentamycin ciprofloxacin neomycin penicillin Metronidazole Ceftazidime Vancomycin streptomycin Bowel lavage: NA	NA	7	200 μL	Feces from normal mice treated with saline by intraperitoneal injection and fed *ad-libitum* or fasting- mimicking diet	Zhou et al., 2019

##### FMT studies in patients (Table 3)

There is only one case report (Huang H. et al., 2019) describing a PD patient that received FMT in whom temporary improvement of leg tremors and other PD symptoms was observed 1 week after three FMTs. Unfortunately, leg tremors recurred 2 months post-FMT and other PD symptoms had also returned to baseline levels 1 month later. On the other hand, constipation had also improved and this improvement lasted until the end of follow-up 3 months post-FMT. No further studies on FMT in PD were identified, except for one communication in a divulgative magazine in which improvement of PD symptoms after FMT was mentioned without further details (Ananthaswamy, 2011).

On ClinicalTrials.gov, one RCT and one non-randomized trial with FMT in PD patients are registered as ongoing trials, and one placebo-controlled RCT with PRIM-DJ2727, an orally administered lyophilized fecal microbiota product, is planned (Appendix 2).

#### Epilepsy

##### Role of the gut microbiota in disease symptoms and pathogenesis

In epilepsy, both genetic and environmental factors are thought to be involved in individual predisposition, but exact etiology of most cases remains unknown. A link between the gut microbiota and the pathophysiology of epilepsy has been proposed by some studies.

A difference in gut microbiota profiles between patients with different types of therapy refractory epilepsy and healthy controls was found in several studies. All these studies reported increased abundance of the phyla Firmicutes relative to Bacteroidetes in subjects with refractory epilepsy (Xie et al., 2017; Peng et al., 2018; Lindefeldt et al., 2019). Some bacteria of the phylum Firmicutes may alter neurotransmitter levels (Peng et al., 2018). Further microbiota analysis outcomes differed considerably between these studies, including α-diversity measures (Xie et al., 2017; Peng et al., 2018; Lindefeldt et al., 2019). One study (Peng et al., 2018) found an increased Firmicutes/Bacteroidetes ratio and α-diversity in drug-resistant patients compared to drug-sensitive patients, with the latter being similar to healthy controls. Importantly, α-diversity was probably increased due to the abnormal increased abundance of rare bacteria. On genus level, several differences were also found. Based on these results, one could hypothesize a role for bacteria in the effectivity of medication for epilepsy, but no causal statements can be made (Peng et al., 2018). Interestingly, zonisamide, an anticonvulsant drug, is metabolized by gut bacteria (Kitamura et al., 1997). Furthermore, an increase in *Bifidobacteria* and *Lactobacillus* was correlated with four or less seizures per year (Peng et al., 2018). Another important finding in patients with epilepsy is that a ketogenic diet reduces the number of seizures and that a ketogenic diet is associated with an altered gut microbiota composition and function (Dahlin and Prast-Nielsen, 2019).

Sewal et al. (2017) found increased seizure susceptibility after intraperitoneal administration of LPS in rats, which was accompanied by increased blood-brain barrier permeability and increased levels of pro-inflammatory cytokines in the brain. Furthermore, contrasting results on whether antibiotic treatment provides protective or inducing effects on seizures are observed in animal and human studies (Lum et al., 2019). Importantly, potential direct neurotoxic effects of the antibiotics themselves or pro-epileptogenic effects of the underlying disease (e.g., infection) that is treated might rather be involved. Furthermore, some studies found a positive effect of probiotics in epilepsy (Gomez-Eguilaz et al., 2018; Yeom et al., 2019).

##### FMT studies in animal models (Table 4)

Medel-Matus et al. (2018) found that transfer of feces from a stressed rat donor increased progression and duration of kindled seizures (i.e., rearing, with or without falling) in sham-stressed rats. The pro-epileptic effects were counteracted in stressed recipients of donor feces from sham-stressed rats. Another study (Olson et al., 2018) observed that a germ-free temporal lobe epilepsy mouse model did not show ketogenic diet-mediated seizure protection. They also observed that the seizure threshold in specific-pathogen-free mice increased after transplantation with ketogenic diet microbiota or long-term administration of species *Akkermansia muciniphila, Parabacteroides merdae*, and *P. distasonis* (associated with a ketogenic diet).

**Table 4:** FMT in epilepsy.

**Study design**	***N***	**Follow-up after FMT**	**Neurological effects of FMT**	**GI effects of FMT**	**FMT-effects on microbiota**	**SAE after FMT (animals: other important effects)**	**Pre-treatment**	**Administration route**	**No. of FMT**	**Amount of feces**	**Rationally selected feces donor**	**References**
Human Case report of generalized epilepsy	1	20 m	More than 20 m seizure-free without antiepileptic drugs.	Crohn's disease: Decrease of CDAI score (361 pre-FMT, 104 at 12 m post-FMT, remained decreased until end of follow-up).	NA	NA	AB: NA Bowel lavage: NA	Gastroscopy under anesthesia	3	200 mL	No	He et al., 2017
Animal model: Kindled seizures (rearing, with or without falling) by kindling of the basolateral amygdala and induction of chronic restraint stress in SD rats Relevant groups (all SD rats): FMT: 1) Stress+sham-FMT 2) sham-stress+stress-FMT No FMT: 3) Sham-stress 4) Stress	6 per group	14 d	In Group 2 and 4 accelerated kindling. In group 1 kindling progressed slower than group 4, comparable to group 3. Increased seizure duration in group 4 vs. 3, prevented in group 1. In group 2 seizure duration comparable to group 4.	NA	NA	NA	AB: vancomycin Neomycin metronidazole ampicillin (ampho-B) Bowel lavage: NA	Oral Gavage	3	2 mL	Pooled feces from stressed or sham-stressed SD rats	Medel-Matus et al., 2018
Animal model: 6-Hz-induced seizure mouse model of refractory temporal lobe epilepsy Relevant groups (all SW mice): FMT: 1) SPF-CDi+CDi-FMT 2) SPF-KDi+CDi-FMT 3) SPF-CDi+KDi-FMT 4) GF-KDi+SPF-FMT No FMT: 5) SPF-KDi 6) GF-KDi 7) SPF-CDi	5–18 per group	Seizure testing 14 d after FMT in exp 1, and 3 d in exp 2.	6-Hz Psychomotor Seizure Assay: Experiment 1: increased seizure threshold in group 4 vs. group 6 and 7, comparable to group 5. Experiment 2: Increased seizure threshold in group 2 and 3 vs. 1.	NA	NA	NA	AB: vancomycin neomycin metronidazole ampicillin Bowel lavage: NA	Oral Gavage	1	100 μL	Donor mice fed CDi or KDi (SPF Swiss Webster mice)	Olson et al., 2018

##### FMT studies in patients (Table 4)

There is one case report (He et al., 2017) of a patient with generalized epilepsy and Crohn's disease that received three FMTs. Before FMTs, she experienced frequent seizures when not using sodium valproate treatment, and after FMTs, the patient was seizure-free without antiepileptic drugs for 20 months. Furthermore, the Crohn's disease activity index improved (He et al., 2017).

One registered interventional study with a single group assignment is ongoing with FMT in patients with epilepsy (Appendix 2).

#### (Diabetic) Neuropathic Pain

##### Role of the gut microbiota in disease symptoms and pathogenesis

Neuropathic pain is pain that is caused by damage (e.g., nerve trauma or chemotherapeutic damage) or diseases (e.g., diabetes mellitus) of the peripheral or central somatosensory nervous system. It is characterized by abnormal sensations or pain following normally non-painful stimulation (Guo et al., 2019). A potential complication of diabetes mellitus is peripheral neuropathy with accompanying neuropathic pain and peripheral neuropathy is positively associated with insulin resistance (Han et al., 2015). Interestingly, patients with diabetes mellitus have a different gut microbiota composition and function compared to controls (Qin et al., 2012; Karlsson et al., 2013; Jamshidi et al., 2019). FMT may alter insulin resistance and thereby neuropathic pain. An increase in insulin resistance was observed in germ-free wild-type mice after FMT with feces from conventionally raised mice (Backhed et al., 2004). In humans, FMT with feces from lean donors in subjects with metabolic syndrome led to increased insulin sensitivity (Vrieze et al., 2012).

The gut microbiota may also regulate pain by directly modulating neuronal excitability of dorsal root ganglia or indirectly by regulating neuroinflammation in the peripheral and central nervous system (Guo et al., 2019). Microbiota depletion by antibiotics or the complete absence of gut microbiota in mice had a protective effect on pain in oxaliplatin-induced peripheral neuropathy, accompanied by decreased infiltration of macrophages and cytokines in the dorsal root ganglia. The effect could be reversed by gut microbiota restoration and this suggests an influence of the gut microbiota on neuropathic pain (Shen et al., 2017). Another study demonstrated a positive effect of probiotics *in vitro* on paclitaxel-induced neuropathic pain features (Castelli et al., 2018). However, when probiotics *L. reuteri* LR06 or *Bifidobacterium* BL5b were administered to rats with chronic constriction injury-induced neuropathic pain, no effect on pain sensation was observed (Huang J. et al., 2019).

##### FMT studies in animal models (Table 5)

One study (Yang C. et al., 2019) observed an increased pain-like phenotype in antibiotic-treated mice. Furthermore, mice that received antibiotics and FMT with feces from a neuropathic pain rat model with anhedonia-like phenotype developed more pain-symptoms compared to antibiotic- and PBS-treated mice. Mice that received antibiotics and non-anhedonia microbiota showed less pain-symptoms compared to PBS-treated mice, comparable to mice receiving feces from sham-operated rats.

**Table 5:** FMT in (diabetic) neuropathic pain.

**Study design**	***N***	**Follow-up after FMT**	**Neurological effects of FMT**	**GI effects of FMT**	**FMT-effects on microbiota**	**SAE after FMT (animals: other important effects)**	**Pre-treatment**	**Administration route**	**No. of FMT**	**Amount of feces**	**Rationally selected feces donor**	**References**
Human (diabetic neuropathy) Case report	1	3 months between FMTs, time of follow-up unclear	Limb pain + paresthesia reduced (VAS from 7.2 to 2.5), without analgesics. Improvement of motor conduction velocity in tibial nerve, without improvement of sensory dysfunction.	NA	NA	Temporary mild AE (nausea, vomiting, diarrhea). Blood glucose decreased and stabilized.	AB: NA Bowel lavage: unclear	Colonoscopy under anesthesia	2	NA	No	Cai et al., 2018
Animal model: Mice that received feces from rat model of SNI (neuropathic pain), with or without anhedonia-like phenotype based on hierarchical cluster analysis of SPT Relevant groups: FMT (AB-induced pseudo-GF WT mice): 1) Anh-FMT 2) Non-anh-FMT 3) Sham-FMT No FMT (WT mice): 4) AB+PBS-FMT 5) Control	7–10 per group per analysis.	6 d	Pain (MWT and TFT): pain-scores increased after AB. Further increased in group 1, vs. group 2, 3 and 4. Decreased pain in group 2 vs. 4, comparable to group 3.	NA	α-diversity: lower in group 4, vs. group 1, 2 and 5. Higher in group 2, vs. group 1, comparable to group 5 (Shannon). β-diversity: different composition of group 4 vs. group 1, 2 and 5 (PCoA). Difference in individual taxa: yes.	Depression-like behavior (FST, TST, SPT): increased after AB before FMT, which was more increased in group 1 and decreased in group 2. Decreased depression-symptoms in group 2 vs. 4, comparable to group 3.	AB: ampicillin neomycin sulfate metronidazole Bowel lavage: NA	Gavage	14	1 g of feces, 0.2 mL of suspension	45 rats with induced SNI (neuropathic pain), either anhedonia susceptible or resilient, or sham-operated rats	Yang C. et al., 2019

##### FMT studies in patients (Table 5)

In a case report, a woman with poorly regulated type 2 diabetes mellitus and diabetic neuropathy experienced improvement of limb pain and paresthesia after two FMTs. Visual analog scale (VAS) pain score decreased. There was improvement of motor conduction velocity in the tibial nerve without improvement of sensory dysfunction on electromyogram. Furthermore, fasting blood glucose levels decreased and stabilized, and HbA1c decreased from 7.5 to 6.3. However, length of follow-up was unclear and therefore it is unknown how long the improvements lasted (Cai et al., 2018).

#### Tourette Syndrome

##### Role of the gut microbiota in disease symptoms and pathogenesis

Tourette syndrome (TS) is a neurodevelopmental disorder characterized by the presence of motor and phonic tics with onset during childhood. It is considered to be caused by an interplay between genetic, environmental and social factors. Reports on an association of the gut microbiota with TS or tic disorders in general are scarce. Liao et al. (2019) observed a decrease of tic-like behaviors in a rat model after administration of *Lactobacillus plantarum* PS128 which coincided with improved dopamine metabolism and norepinephrine levels in the striatum and prefrontal cortex. A study of 30 patients with pediatric acute-onset neuropsychiatric syndrome (PANS) and pediatric autoimmune neuropsychiatric disorders associated with streptococcal infections syndrome (PANDAS), in which tic disorders may appear following a streptococcal infection, revealed a different gut microbiota composition compared to healthy controls. In addition, a decrease in pathways involved in brain function and an increase of some pathways involved in modulation of the antibody response to intestinal inflammation were observed in younger PANS/PANDAS patients, although the sample size was small (Quagliariello et al., 2018). Another study (Snider et al., 2005) found that penicillin plus azithromycin prophylaxis was effective in decreasing streptococcal infections and the associated neuropsychiatric disorders, including tic disorders, in PANDAS patients. However, the role of the gut microbiota is unclear in this case, as the improvement of the neuropsychiatric symptoms could also be related simply to the concomitant suppression of streptococcal infections. Moreover, the sample size was small and the applied methodology was questionable (Budman et al., 2005).

In an open-label clinical trial, described in an abstract (Ding et al., 2019), a transient decrease of tic severity was observed in 11 males with TS after treatment with three administrations of a mixed bacterial community.

##### FMT studies in animal models

No animal studies on FMT and TS were identified.

##### FMT studies in patients (Table 6)

Zhao H. et al. (2017) described a case report of a child with TS that had decreased tic severity at the follow-up moment 8 weeks after FMT. The parents reported that they had observed disappearance of involuntary phonation, a decrease in involuntary shrugging and improved attention in the 8 weeks after FMT. Further follow-up was not described.

**Table 6:** FMT in Tourette syndrome.

**Study design**	***N***	**Follow-up after FMT**	**Neurological effects of FMT**	**GI effects of FMT**	**FMT-effects on microbiota**	**SAE after FMT (animals: other important effects)**	**Pre-treatment**	**Administration route**	**No. of FMT**	**Amount of feces**	**Rationally selected feces donor**	**References**
Human Case report	1	8 w	YGTSS scores:Total tic: decreased from 31 to 5, motor: decreased from 16 to 5, vocal: decreased from 15 to 0. Report parents: severity tic symptoms ameliorated,involuntary phonation disappeared, involuntary shrugging decreased, more focused.	NA	NA	No AE during FMT. Long-term post-FMT AE unclear.	AB: NA Bowel lavage: NA	Small intestine via gastroscopy and colon via colonoscopy under anesthesia	1 via gastroscopy, 1 via colonoscopy	Gastroscopy: 100 mL, colonoscopy: 300 mL	No	Zhao H. et al., 2017

### Neurological Disorders With FMT Studies Only in Animal Models

#### Stroke

##### Role of the gut microbiota in disease symptoms and pathogenesis

In some studies, patients with stroke appeared to have an altered gut microbiota composition compared to healthy controls (Karlsson et al., 2012; Yin et al., 2015), although some other studies reported that no change occurred or that change was only temporary (Koren et al., 2011; Swidsinski et al., 2012). However, these results may be confounded by factors, such as age, type 2 diabetes mellitus or obesity, which are risk factors for stroke that are associated with a different gut microbiota composition (Qin et al., 2012; Karlsson et al., 2013; Torres-Fuentes et al., 2017; An et al., 2018). For α-diversity, studies are also contradictory (Yin et al., 2015; Li N. et al., 2019). Furthermore, decreased neuronal injury and improved cognitive performance was observed in diabetic mice with bilateral common carotid arteries occlusion after receiving *Clostridium butyricum* suspension intragastrically (Sun et al., 2016).

Studies on the role of the gut microbiota in stroke are frequently contradictory. It has been hypothesized that the gut microbiota influences the severity of ischemic brain injury following stroke. After stroke, reduced intestinal motility combined with reduced α-diversity of bacterial species, bacterial overgrowth and impaired intestinal barrier may lead to formation of proinflammatory immune cells in the gut-associated lymphoid tissue and subsequent infiltration of the brain with increased infarct volume (Singh et al., 2016). Possibly, the translocation of gut bacteria and their metabolites is also involved (Caso et al., 2009; Chen et al., 2019). However, gut microbiota depletion with bacterial outgrowth of certain taxa and reduced α-diversity may also have a protective effect on ischemic brain injury by suppression of trafficking of effector T cells from gut to brain (Benakis et al., 2016). The immune function appears to be reduced after stroke, with impairment of the gut-associated lymphoid tissue (Meisel et al., 2005; Schulte-Herbruggen et al., 2009). Furthermore, the reduced intestinal motility in stroke patients is reflected by the increased frequency of constipation (Camara-Lemarroy et al., 2014).

Another role of the gut microbiota may be hypothesized in the development of atherosclerosis and subsequent stroke. Several studies in animal models and humans suggest that the gut microbiota influences the formation of atherosclerotic plaques (Stepankova et al., 2010; Koren et al., 2011; Wang Z. et al., 2011; Gregory et al., 2015; Zhu et al., 2016). Symptomatic atherosclerosis in humans is indeed associated with a different gut microbiota composition and functional capacity (Karlsson et al., 2012). Production of trimethylamine-N-oxide by the gut microbiota may be associated with cardiovascular events, including stroke (Nam et al., 2019; Yang S. et al., 2019), whereas others studies suggest a protective effect (Yin et al., 2015; Collins et al., 2016; Arduini et al., 2019). Nevertheless, atherosclerosis is not the only determinant of stroke.

##### FMT studies in animal models (Table 7)

Winek et al. (2016) found similar infarct volumes but increased mortality in temporarily antibiotic-treated mice after stroke induction compared to stroke mice without antibiotics or continuous antibiotics and sham-operated antibiotic-treated mice. Temporarily antibiotic-treated mice also developed severe colitis. When these mice received gavage with specific-pathogen-free microbiota before stroke-induction, a similar mortality and infarct volume was observed, but sample size was low. However, colitis was prevented. This means colitis could not have caused the increased mortality in the FMT-group. Antibiotics may have played a role as mortality in the FMT-group was similar to the antibiotic treated-stroke group (Winek et al., 2016). Two other studies found more functional impairment, larger cerebral infarct volume, and increased intestinal, systemic and cerebral inflammation in (pseudo-)GF stroke mice that had received dysbiotic post-stroke mouse or human microbiota compared to mice receiving normal microbiota (Singh et al., 2016; Xia et al., 2019). In addition, gavage with normal microbiota led to reduced infarct volumes (Singh et al., 2016). In contrast, Benakis et al. (2016) found a protective effect of microbiota depletion by antibiotics on infarct volume. Reduced ischemic brain injury and sensorimotor deficits were observed in amoxicillin/clavulanic acid (AC)-sensitive mice treated with AC compared to AC-resistant mice. AC-treated mice that had received AC-sensitive microbiota revealed a decreased infarct volume compared to mice receiving AC-resistant microbiota.

**Table 7:** FMT in stroke.

**Study design**	***N***	**Follow-up after FMT**	**Neurological effects of FMT**	**GI effects of FMT**	**FMT-effects on microbiota**	**SAE after FMT (animals: other important effects)**	**Pre-treatment**	**Administration route**	**No. of FMT**	**Amount of feces**	**Rationally selected feces donor**	**References**
Animal model: Mice with MCAO-induced transient focal cerebral ischemia Relevant groups (all SPF WT mice): FMT: 1) AB (+/–) MCAO+SPF-FMT 2) AB (+/–) sham+SPF-FMT No FMT: 3) AB (+/–) MCAO 4) AB (+/+) MCAO 5) AB (+/–) sham 6) AB (+/+) sham 7) Short-AB (+/–) MCAO 8) MCAO control 9) control	5–25 per group per analysis	11 d (MCAO/ sham 4 d after FMT)	No difference in infarct volume 1 d after MCAO between MCAO-groups. 3/7 died in group 1 and 4/6 in group 3. Mortality in group 1 higher than in group 8 (0/8) and no significant difference with group 2, 3, 4, and 6. Mortality in group 2 lower than group 3 and no significant difference with group 1, 4, 6, and 8.	No colitis in group 1, 2, 4, 6, 7, and 9.Colitis in group 3 and 5 (AB-induced).	α-diversity: NA. β-diversity: NA. Difference in individual taxa: restoration of main bacterial groups after FMT.	NA	AB: ampicillin vancomycin ciprofloxacin imipenem metronidazole Bowel lavage: NA	Oral gavage	2	0.3 mL supernatant of fecal suspension	Feces from SPF littermates without antibiotics or surgery	Winek et al., 2016
Animal model: Mice with MCAO-induced transient focal cerebral ischemia Relevant groups: FMT (SPF WT mice): 1) AC Res FMT+ MCAO 2) AC Sens FMT+ MCAO	8 per group per analysis	17 d (MCAO 2 w after FMT)	Infarct volume (72 h post MCAO) reduced by 54 ± 8% in group 2 vs. 1.	NA	α-diversity: lower in group 2 vs. 1 (Shannon). β-diversity: NA. Difference in individual taxa: yes.	NA	AB: pulse treatment with AC for 3 d Bowel lavage: NA	Oral gavage	1	200 μL	Pooled cecal contents of mice with AC-resistent or AC-sensitive microbiota (post-FMT mice kept on water until MCAO)	Benakis et al., 2016
Animal model: Mice with induced focal cerebral ischemia by: 1) cMCAO OR 2) fMCAO Relevant groups: FMT (GF WT mice): 1) fMCAO-FMT+ cMCAO 2) Sham-FMT+ cMCAO 3) fMCAO+WT-FMT 4) Rag 1–/–: fMCAO+WT-FMT No FMT (WT mice): 5) sham 6) fMCAO+vehicle 7) Rag 1–/–: fMCAO+ vehicle	5–8 per group per analysis	Model 1: 8 d (cMCAO 3 d post-FMT). Model 2: 3 d (FMT on stroke-induction day).	Model 1: Group 1 vs. 2: - Cylinder test: more functional impairment. - Larger infarct size. - Increased expression of pro-inflammatory IL-17 and IFN-γ in brains. - No difference in cerebral Foxp3. Model 2: Group 3 vs. 6: - Reduced infarct volume. - More Foxp3+ Treg in brain (and spleen). - Rag1–/–: no effect of FMT on infarct size.	Model 1: Group 1 vs. 2: Increased CD11b+ monocytes in terminal ileum. Increased Th1- and Th17-cells in Peyer's patches (migrate to infarct area in brain).	Model 1: α-diversity: NA. β-diversity: changed, more like donors (Bray-Curtis). Difference in individual taxa: NA. Model 2: α-diversity: group 3 higher than 6 and similar to 5 (Shannon). β-diversity: NA. Difference in individual taxa: yes.	NA	AB: NA Bowel lavage: NA	Gastric gavage	M1: 1, M2: Daily from stroke induction until death	200 μL	Model 1: Cecum microbiota from sham or fMCAO donor mice Model 2: Feces from SPF WT mice	Singh et al., 2016
Animal model: Mice with MCAO-induced transient focal cerebral ischemia. Relevant groups: FMT (SPF WT mice): 1) A+Y-FMT (+MCAO) 2) Y+Y-FMT (+MCAO) 3) A+Y-FMT (+sham) 4) Y+Y-FMT (+sham) 5) Y+A-FMT (+MCAO) 6) A+A-FMT (+MCAO) 7) Y+A-FMT (+sham) 8) A+A-FMT (+sham) 9) 24:A-FMT+MCAO, 10) 24:A-FMT+sham, 11) 24:Y-FMT+MCAO, 12) 24:Y-FMT+sham	2–16 per group per analysis	2 m (MCAO + reperfusion 1 m post-FMT)	Group 1 and 2 vs. group 5 and 6: - Improved NDS, HWT and OFT (no difference in sham groups) - Decreased infarct size (only in aged recipient mice, and overall increased relative infarct volume in young recipient mice) - Decreased mortality.	NA	α-diversity: NA. β-diversity: NA. Difference in individual taxa: yes. Group 1 and 2 vs. group 5 and 6: Increased SCFA in feces.	Group 11 vs. group 9 (no difference in sham groups): increased protective cytokines (IL-4+G-CSF) and reduced pro-inflammatory cytokines (TNF-α, eotaxin, CCL5) in plasma.	AB: streptomycin Bowel lavage: NA	Gavage	5	50 μL	Pooled feces from 3 young (8–12 w old) or pooled feces from 3 old (18–20 m old) mice	Spychala et al., 2018
Animal model: Mice with MCAO-induced transient focal cerebral ischemia Relevant groups: FMT (WT AB-induced pseudo-GF mice): 1) SDI-H-FMT+MCAO 2) SDI-L-FMT+MCAO	10 per group in total, 4-9 per group per analysis	17 d from 1st FMT	Group 1 vs. 2: increased infarct volumes and exacerbated neurological functional impairment (mNSS). Overall mortality rate 10% in both FMT-groups.	Group 1 vs. 2: more (IL-17+) γδ T cells among intestinal intraepithelial lymphocytes of the small intestine.	α-diversity: NA. β-diversity: group 1 different gut microbiotathan 2 (unw. UniFrac+PCoA). Four genera of SDI-H successfully transplanted in group 1. Difference in individual taxa: yes.	In group 1 increased pro-inflammatory (IL-17+) γδ T cells and reduced (CD4+CD25+) T cells in the spleen.	AB: pulse-treatment: vancomycin metronidazole gentamycin ampicillin Bowel lavage: NA	gavage	14	0.2 mL	3 humans with high SDI or low SDI	Xia et al., 2019

Another study found that gut microbiota in young mice is altered after experimental stroke and resembles that of uninjured aged mice. They found improved performance in several behavioral tests, decreased mortality and infarct size and decreased pro-inflammatory cytokines after FMT with young microbiota compared to those receiving aged microbiota (Spychala et al., 2018).

#### Alzheimer's Disease

##### Role of the gut microbiota in disease symptoms and pathogenesis

Alzheimer's disease (AD) is a neurodegenerative disease with a progressive decline in cognitive function and loss of neurons and synapses. A combination of genetic and environmental factors is thought to be involved in the etiology (Angelucci et al., 2019). Pathology is characterized by intraneuronal deposits of neurofibrillary tangles and extracellular accumulations of abnormally folded amyloid beta (Aβ) proteins. Aβ proteins are thought to be pro-inflammatory neurotoxic proteins (Calsolaro and Edison, 2016). However, the hypothesis that Aβ proteins depositions lead to synaptic dysfunction and subsequently symptoms has been questioned (Selkoe and Hardy, 2016). It is unclear whether Aβ protein deposition may be the cause or result of AD. There could be a vicious cycle between Aβ accumulation leading to microglia activation and microglia dysfunction leading to Aβ accumulation (Cai et al., 2014). Neuroinflammation is thought to play a key role in AD (Calsolaro and Edison, 2016).

In recent years, numerous publications on the relation between AD and the gut microbiota have become available. AD patients have a different gut microbiota composition compared to healthy controls or elderly without dementia (Vogt et al., 2017; Zhuang et al., 2018; Haran et al., 2019; Li B. et al., 2019; Liu et al., 2019), but α-diversity measures show contrasting results (Vogt et al., 2017; Li B. et al., 2019; Liu et al., 2019; Saji et al., 2019). Hypotheses on the role of the gut microbiota include direct actions of bacteria, indirect actions or aging-related processes (Angelucci et al., 2019).

Studies have demonstrated the presence of microorganisms in brains of both AD patients and healthy controls. However, increased LPS levels (Zhan et al., 2016; Zhao Y. et al., 2017) and an increased presence of several bacteria and fungi, including some gut commensals, were observed in brains of AD patients compared to controls (Mawanda and Wallace, 2013; Pisa et al., 2017; Alonso et al., 2018; Dominy et al., 2019). The presence of bacterial LPS or endotoxin-mediated inflammation contributes to amyloid neurotoxicity (Zhao et al., 2015). A study found that LPS in the brain colocalizes with AD-related Aβ proteins in amyloid plaques (Zhan et al., 2016). Interestingly, the LPS- and microorganism-detecting receptor CD14, important in the neutralization of invading microorganisms, is also stimulated by Aβ fibrils (Zhao et al., 2015). Furthermore, feces of patients with brain amyloidosis and cognitive impairment contain more pro-inflammatory gut bacteria and blood more pro-inflammatory cytokines compared to patients with cognitive impairment without amyloidosis or controls. In addition, less anti-inflammatory bacteria and cytokines are observed (Cattaneo et al., 2017). Interestingly, certain bacteria can produce extracellular bacterial amyloids known as “curli fibers,” a main component of biofilms for these bacteria. Some bacteria that produce these curli fibers are recognized by the same Toll-like receptor as the Aβ42 peptide that accumulates in AD (Zhao et al., 2015; Tursi and Tükel, 2018). Increased levels of *E. coli*, a curli fiber producer, were indeed found in AD brains compared to controls (Zhan et al., 2016). However, it is unknown whether bacterial amyloids co-localize with the amyloid deposits or other insoluble lesions that are observed in AD (Zhao et al., 2015; Tursi and Tükel, 2018). Bacteria may also contribute to AD by production of neurotransmitters and altering proteins and receptors involved in synaptic plasticity (Barrett et al., 2012; Maqsood and Stone, 2016).

Apart from the direct action of bacteria, it is suggested that certain gut microbiota alterations may stimulate inflammatory pathways and thereby neuroinflammation (Marques et al., 2016). Some researchers hypothesized that Aβ depositions are part of an innate immune response that normally protects against microbial infections in the brain (Moir et al., 2018). A decrease of Aβ accumulation and microglia activation after antibiotic treatment has indeed been observed. Conversely, other studies suggested that microbiota depletion may negatively affect cognitive function and microglia and synaptic function in mice and contrasting results were also observed in humans (Laake and Oeksengaard, 2002; Angelucci et al., 2019). Furthermore, a positive effect of probiotics on cognitive function was observed in animal models and AD patients or adults with mild cognitive impairment (Davari et al., 2013; Jiang et al., 2017; Kobayashi et al., 2017, 2019a; Rezaei Asl et al., 2019; Tamtaji et al., 2019), whereas one study reported no to little effect (Kobayashi et al., 2019b).

Erny et al. (2015) observed defects of microglia in germ-free mice leading to impaired innate immune responses. This is in line with the hygiene hypothesis, that proposes that AD patients have reduced microbial diversity due to environmental sanitation and therefore an impaired response to pathogens, with an important role for T cells, in particular Treg cells (Larbi et al., 2009; Browne et al., 2013; Dansokho et al., 2016; Hu et al., 2016). Furthermore, with increasing age, an increase in proinflammatory cytokines and decrease in anti-inflammatory gut bacteria is observed (Biagi et al., 2010). This inflamm-aging may also be associated with cognitive decline (Franceschi et al., 2000).

##### FMT studies in animal models (Table 8)

Zhan et al. (2018) observed that wild-type mice showed decreased cognitive function after broad-spectrum antibiotic treatment. Subsequently, spatial learning and memory improved after receiving feces from senescence-resistant mice (similar to control) compared to mice receiving feces from senescence-prone mice (similar to antibiotics plus vehicle). Using diabetic mice with and without cognitive deterioration gave a similar result (Yu et al., 2019), although this is not a mouse model of AD but cognitive deterioration secondary to diabetes. These two studies suggest that antibiotics decrease cognitive function in mice, which may be reversed by FMT. In contrast, two studies (Harach et al., 2017; Dodiya et al., 2019) observed reduced Aβ-pathology and neuroinflammation in male mice (not in female mice) when the gut microbiota was depleted. When an AB-treated AD mouse model received FMT with feces from age- and sex-matched AD mice without antibiotics, the positive effect of antibiotics was partially reversed (Dodiya et al., 2019). In a germ-free AD mouse model, FMT with conventional microbiota or conventional AD microbiota caused an increase of pathology, with the latter showing a stronger effect (Harach et al., 2017). Another study with germ-free wild-type mice found a deterioration of cognitive function with lower fecal metabolites related to the nervous system, such as GABA, in aged, and not young, mice that received AD feces compared to healthy control feces (Fujii et al., 2019).

**Table 8:** FMT in Alzheimer's disease.

**Study design**	***N***	**Follow-up after FMT**	**Neurological effects of FMT**	**GI effects of FMT**	**FMT-effects on microbiota**	**SAE after FMT (animals: other important effects)**	**Pre-treatment**	**Administration route**	**No. of FMT**	**Amount of feces**	**Rationally selected feces donor**	**References**
Animal model: APPPS1 transgenic mice Relevant groups: FMT (GF APPPS1 mice): 1) cAPPPS1-FMT 2) cWT-FMT No FMT: (APPPS1 mice): 3) GF APPPS1 4) cAPPPS1	4–6 per group per analysis	8 w after 1st FMT.	In 6 m old mice: higher cerebral Aβ38, Aβ40 and Aβ42 levels in group 1, 2, and 4 vs. group 3. Higher cerebral Aβ40 and Aβ42 levels in group 1 and 4 vs. group 2. Plasma Aβ42 levels increased in group 1, 2 and 3 vs. group 4 and no difference in plasma Aβ40 levels. Not significant decrease in Aβ degrading enzymes in group 1 and 2 vs. group 3.	NA	α-diversity: higher in group 2 vs. 1, on d of FMT, which disappears later (Chao1). β-diversity: probably different between group 1 and 2 (w. Unifrac+PCoA), Difference in individual taxa: yes.	NA	AB: NA Bowel lavage: NA	Oral gavage	2	0.2 mL	Pooled feces from aged APPPS1 mice with conventional microbiota or aged (12 m old) WT mice with conventional microbiota	Harach et al., 2017
Animal model: SAMP8 miceRelevant groups: (young SAMP 8 mice) FMT: 1) SAMP8-HN-FMT 2) SAMP8-FMT	6 per group	30 d after 1st FMT	Increased cerebral Aβ in group 1 vs. group 2. Decreased tight junction proteins in brain in group 1 vs. group 2 (CLDN1 and ZO-1).	Decreased tight junction proteins in intestines in group 1 vs. group 2 (CLDN1 and ZO-1).	NA	NA	AB: NA Bowel lavage: NA	Oral gavage	8	100 μL	SAMP8 control mice and SAMP8 mice exposed to HN	Cui et al., 2018
Animal model: Mice that received feces from SAMP8 mice Relevant groups: FMT (AB-induced pseudo-GF WT mice): 1) SAMP8-FMT 2) SAMR1-FMT No FMT (WT mice): 3) Control 4) Vehicle (pseudo-GF)	7–8 per group per analysis	21 d from first FMT.	Escape latency and escape path length (spatial learning) and tests in probe trial (spatial memory): better performance in group 2 vs. group 1; similar performance in group 1 and 4; similar performance in group 2 and 3.	NA	α-diversity: Increased in group 2 vs. 1, and Decreased in group 4 vs. 3. Group 4 and 1 similar (Chao1, observed species index, Phylog. div. whole tree, Shannon). β-diversity: different in all groups (Bray-Curtis+PCoA). Difference in individual taxa: yes.	NA.	AB: ampicillin neomycin sulfate metronidazole Bowel lavage: NA	Gavage	14	1 g of feces, 0.2 mL fecal suspension	Feces from SAMR1 or SAMP8 mice	Zhan et al., 2018
Animal model: Mice that received feces from a streptozotocin-induced T1D mouse model with or without CD (hierarchical cluster analysis of MWMT performance) Relevant groups: FMT (AB-induced pseudo-GF WT mice): 1) T1D-CD-FMT 2) T1D-Non-CD-FMT No FMT (WT mice): 3) Vehicle (pseudo-GF) 4) Control	7–8 per group per analysis	21 d from first FMT.	Escape latency (spatial learning) and tests in probe trial (spatial memory): better performance in group 2 vs. 1; similar performance in group 1 and 3; similar performance in group 2 and 4. No differences in escape path length.	NA	α-diversity: No differences between groups (Shannon +Simson). β-diversity: different in all groups (PCoA, OTU-based PLS-DA analysis and unw. Unifrac). Difference in individual taxa: yes.	NA	AB: ampicillin neomycin sulfate metronidazole Bowel lavage: NA	Gavage	14	1 g of feces, 0.2 mL fecal suspension	Feces from CD or non-CD mice	Yu et al., 2019
Animal model: APPPS1-21 mouse model Relevant groups (all AB-treated APPPS1-21 male mice): FMT: 1) APPPS1-21-FMT No FMT: 2) Vehicle	8–9 per group per analysis	24 d after 1st FMT (until end of all FMT)	Group 1 vs. 2: More Aβ-burden, larger plaque size in the cortex and larger microglia cell body area. No changes in total microglia numbers. Shortening of dendritic branch lengths and reduction of dendritic branch points in microglia.	Lower cecum weight in group 1 vs. 2.	α-diversity: lower in group 2 vs. donor; group 1 no differenceswith donor (Pielou's evenness + FPD). β-diversity: difference between donor and group 2 and a difference between group 1 and group 2. Clustering of donor and group 1 (unw. Unifrac+ PCoA). No difference in w. UniFrac. Difference in individual taxa: yes.	NA	AB: kanamycin gentamicin colistin metronidazole vancomycin Bowel lavage: NA	Gavage	24	200 μl fecal suspension	4 SPF control age-matched APPPS1-21 male donor mice	Dodiya et al., 2019
Animal model: Mice that received feces from a human AD patientRelevant groups: FMT (GF WT mice): 1) Human-HC-FMT 2) Human-AD-FMT	7 per group	71 w	OLT and ORT: Deterioration of cognitive function in group 2 vs. 1, starting at the age of 55 w and at several time points (no difference in younger mice).	NA	α-diversity: higher in group 2 vs. 1 (Shannon). β-diversity: mice clustered based on only donor microbiota (w. UniFrac+ PCoA). Difference in individual taxa: yes. Fecal metabolites: GABA, taurine, tryptophan, tyrosine, valine more abundant and propionic acid less abundant in group 1.	NA	AB: NA Bowel lavage: NA	Orally	Probably 1	0.15 mL	AD patient and age-matched HC	Fujii et al., 2019

Cui et al. (2018) found that the chronic noise exposure-associated increased risk for AD in mice may be mediated by the gut microbiota, and that chronic noise causes age-related neurochemical and inflammatory dysregulation. Gavage with feces from high-intensity noise AD mice to age-matched recipient AD mice led to increased cerebral Aβ and affected intestinal and blood-brain barrier compared to control-microbiota recipients.

##### FMT studies in patients

No published FMT-studies in humans with AD were found, but ClinicalTrials.gov showed an ongoing RCT with FMT in patients with AD.

#### Guillain-Barré Syndrome

##### Role of the gut microbiota in disease symptoms and pathogenesis

Guillain-Barré syndrome (GBS) is a paralytic neuropathy, assumed to be caused by an auto-immune response after infection or other immune stimulation, predominantly preceded by gastrointestinal infection with *Campylobacter jejuni*. The disease is frequently characterized by rapidly progressing bilateral weakness. This may be accompanied by additional neurologic or autonomic symptoms. Symptom severity varies among different patients. Eventually, most patients improve, but permanent disability can occur (Willison et al., 2016).

Several studies reported an association between *C. jejuni* colonization/infection and the gut microbiota. *C. jejuni* colonization may be inhibited by gut microbiota-mediated colonization resistance (Brooks and Mansfield, 2017). However, several studies suggest that *C. jejuni* may overcome this colonization resistance in several ways, such as increasing acetinogenesis gene expression, which leads to the conversion of pyruvate to acetate (Ducarmon et al., 2019). Inoculation of mice with *C. jejuni* strains from GBS patients causes less colitis but more autoantibodies with increased peripheral nerve lesions compared to inoculation with *C. jejuni* strains from colitis patients (Malik et al., 2014; St Charles et al., 2017). This is exacerbated after antibiotic treatment (St Charles et al., 2017; Brooks et al., 2019). The production of cross-reactive anti-ganglioside antibodies during *C. jejuni* infection due to molecular mimicry between bacterial LPS of GBS-associated *C. jejuni* strains and peripheral nerve gangliosides have been considered as one of the factors associated with the development of GBS (Jacobs et al., 1997; Yuki, 1997; Ang et al., 2002). This molecular mimicry is more frequently observed in GBS-associated *C. jejuni* strains compared to colitis-associated *C. jejuni* strains.

Antibiotic-treated and gnotobiotic mice display increased colonization and gastrointestinal inflammation after *C. jejuni* inoculation (Chang and Miller, 2006; Stahl et al., 2014; O'Loughlin et al., 2015), with a potential protective role for *Enterococcus faecalis* (O'Loughlin et al., 2015). Furthermore, a considerably decreased *C. jejuni* clearance time is observed in mice that receive antibiotics and gavage with murine microbiota compared to human microbiota-receiving mice and gnotobiotic mice, that showed a more pro-inflammatory immune response (Bereswill et al., 2011).

Moreover, probiotics were successful at reducing *C. jejuni* load in poultry (Morishita et al., 1997; Willis and Reid, 2008). This was also confirmed by one mouse study (Wagner et al., 2009) and one study showed that a probiotic inhibited *C. jejuni* invasion of human intestinal epithelial cells (Wine et al., 2009).

##### FMT studies in animal models (Table 9)

Brooks et al. (2017) observed increased colonization, Th-2 responses (independent of inoculation status) and autoimmune responses after *C. jejuni* injection in a human microbiota-treated mouse group compared to a conventional mouse microbiota group. However, this should be interpreted with caution as *C. jejuni* strains may have been adapted to the human microbiota, while human and animal gut microbiota differ. This study suggests that a particular gut microbiota composition may enhance susceptibility to GBS after *C. jejuni* infection.

**Table 9:** FMT in Guillain-Barré syndrome.

**Study design**	***N***	**Follow-up after FMT**	**Neurological effects of FMT**	**GI effects of FMT**	**FMT-effects on microbiota**	**SAE after FMT (animals: other important effects)**	**Pre-treatment**	**Administration route**	**No. of FMT**	**Amount of feces**	**Rationally selected feces donor**	**References**
Animal model: Mice infected with *C. jejuni* strains from colitis or GBS patients Relevant groups: FMT (SPF or STER WT mice): 1) Hu-260.94 2) Hu-11168 3) Hu-TSB No FMT (SPF WT or IL-10^−/−^ mice): 4) Conv-260.94 5) Conv-11168 6) Conv-TSB	10 per group	5–7 w after *C. jejuni* inoculation.	Group 1 + 2, vs. WT group 4 +/or 5: - OFT: diminished activity - Th1/Th17-dependent anti-*C. jejuni* responses decreased in STER group 2 after 5 w, but increased for SPF (except for Th1) and group 1 after 7 w - Th2-dependent anti-*C. jejuni* responses increased (also elevated in some of TSB-groups) - anti-ganglioside autoantibodies after *C. jejuni* infection elevated after 5 w, less clear after 7 w (also some elevated in group 3).	SPF Group 1+2, vs. WT group 4+/or 5: - *C. jejuni* colonization increased - More pathologic changes in draining lymph nodes, with mild changes in colon or cecum - anti-inflammatory (IL-4) colon responses of group 2 increased after 5 w but not after 7 w - More frequently soft feces. Gross pathology of GI tract observed (thickened cecal and colon wall and/or enlarged draining lymph nodes): in 3/10 STER group 1 and 1/10 STERGroup 2, 4/10 SPF group 1, 5/10 SPF group 2, 5/10 IL-10–/– group 5 mice.	α-diversity: more OTUs in group 5 vs. SPF group 2. No difference in OTUs, chao1, Pielos evenness and Inverse Simpson. β-diversity: Clear separation between Hu- and conv- groups, not affected by inoculation status (PCA, ANOSIM, PERMANOVA). Difference in individual taxa: yes.	- Clinical signs:In SPF group 1 and 2 12/20 with soft feces, hunched posture, rough hair coat and reduced activity compared to 1/20 WT group 4 and 5 with soft feces.1/10 in IL-10–/– group 5 mice.1/10 in group 6 with reduced activity, 2/20 in group 3 with rough hair coat.	AB: NA Bowel lavage: NA	Gavage	NA	NA	Pooled healthy human feces	Brooks et al., 2017

### Ongoing Studies in Other Neurological Disorders

Although no FMT-studies are available yet for amyotrophic lateral sclerosis, one placebo-controlled RCT with FMT in human amyotrophic lateral sclerosis patients is registered on ClinicalTrials.gov.

## Discussion

Neurological disorders are complex, often involving cognitive, motor and systemic aspects. A combination of genetic and environmental factors is mostly thought to be involved in the pathogenesis, with the gut microbiota being one potential factor. Gut microbiota manipulation by FMT may influence symptoms or progression of neurological disorders by gut microbiota-mediated immunological, endocrine, metabolic and/or neural pathways. Gut microbiota-produced metabolites and cytokines may influence the level of intestinal and systemic inflammation and may alter intestinal barrier function. The vagus nerve provides a direct neural connection between the gut and the brain and may play an important role. Furthermore, gut microbiota interventions may influence the availability and efficacy of medication by directly or indirectly interacting with the processing of drugs or by modulation of the immune response.

Gut microbiota manipulation by FMT could be a promising treatment approach for several neurological disorders, but the evidence is limited. The neurological disorder with the most evidence on efficacy of a healthy donor FMT is ASD. Various articles, including clinical trials in humans, suggest that FMT leads to decreased symptom severity. FMT may alter production of gut microbial metabolites, such as serotonin, SCFA or GABA receptor agonists, whereas a reduction of pro-inflammatory gut bacteria may decrease neuro- and systemic inflammation and cerebral oxidative stress. However, the reliability of conclusions in humans might be undermined by the lack of a blind design, as a placebo-effect may play an important role. More and larger double-blind controlled studies are needed. For PD, MS, AD and stroke, several animal studies suggest a positive effect of FMT, supported by some case reports in humans. The potential beneficial effects of FMT for patients with PD could be mediated through a decreased α-syn accumulation in the intestinal wall and subsequently in the brain, by reduced inflammation-induced oxidative stress. Beneficial effects on Parkinson symptoms could be further strengthened by an increased availability and efficacy of levodopa following FMT with feces from donors with less bacterial tyrosine decarboxylases in their feces (Maini Rekdal et al., 2019; van Kessel et al., 2019). Remarkably, one mouse study showed that administration of PD feces is enough to induce PD symptoms (Sun et al., 2018), whereas another study showed that genetic abnormalities in addition to a certain microbiota composition is necessary to induce PD symptoms (Sampson et al., 2016). In MS patients, an increase in Treg cells after FMT may attenuate auto-immunity with demyelination and possibly disease progression. Furthermore, FMT with feces from young healthy donors may decrease AD progression by reducing translocation of pro-inflammatory gut bacteria from gut to brain and consequently neuroinflammation processes mediated by them. However, most animal studies only examined the effects of FMT with feces from AD patients or feces from animal models of AD in healthy mice and not the opposite. The effects of FMT in animal models of stroke are less clear. Performing FMT in patients with high-risk for stroke may potentially decrease infarct size when they develop stroke by a decrease of pro-inflammatory immune cells trafficking to the infarct area. When this mechanism is confirmed by future studies, this suggests that FMT performed immediately after a stroke may also potentially improve infarct size, but no studies have assessed this yet. Interestingly, one mouse study (Winek et al., 2016) reported an increased mortality rate, but similar infarct volume, after FMT. The increased mortality may have been linked to increased infarct size due to FMT, since infarct size was only measured on day 1 after stroke-induction, but it could also be linked to the antibiotic treatment. Remarkably, contrasting effects of microbiota depletion by antibiotics were observed for stroke and AD. The experimental results on FMT for epilepsy are even more difficult to interpret, considering that there are several different subtypes of epilepsy with different pathophysiology. Some, but very limited evidence is available that suggests a potential role of the gut microbiota in seizure susceptibility. Animal studies may imply that the gut microbiota mediates a stress-induced increase and ketogenic diet-induced decrease in seizure susceptibility. For other neurological disorders, such as TS, diabetic neuropathy and Guillain-Barré, the available evidence is very limited. For these disorders and for epilepsy, animal studies need to be replicated and expanded in future studies before an attempt in humans.

Numerous trials on FMT in neurological disorders are currently planned or ongoing and it is expected that the bulk of evidence on the efficacy of FMT in neurological disorders will grow.

For many disorders, research to date has been limited to animal models. It should be noticed that in this field, as in other fields, it is often not known whether results obtained in animal studies are directly applicable to human patients. Robustness of quality and mechanistic depth of animal models differ widely for neurological disorders. Furthermore, behavior and cognition tests in animal models may be subjective or difficult to interpret and not reproducible. Another important consideration is that the immunoreactivity of animal models could be very different from that observed in patients. In some animal studies, feces from humans were transferred to animal models. This may not provide a good model for diseased or healthy humans, considering the differences in gut microbiota and subsequent host-microbiota interactions between humans and animals (Hugenholtz and de Vos, 2018). For this reason, assessment of gut microbiota composition in animal models of disease may also not be reliable. Coprophagy, behavior that is frequently observed in mice (Ebino et al., 1987), may also affect the results of gut microbiota analyses and efficacy of FMT. Several studies attempted to avoid this by caging mice separately, but autologous FMT by coprophagy may still occur. This must all be appraised, when translating findings in animal models into human neurological disorders.

There are also other important limitations on both the human and animal studies with FMT. These limitations are related to the, often complex, study design, including for example the use of different antibiotics, different FMT procedures, the choice of different donors, and the lack of long term follow-up or appropriate control groups.

For human studies, neuropsychiatric diseases analyzed with subjective outcome measures are prone to strong placebo effects after such an invasive treatment (Belcher et al., 2018). A double-blinded design including a control group (for example with autologous FMT) is desirable. Moreover, it is important to acknowledge the possibility of publication bias, especially regarding case reports. Case descriptions and studies with little or no effect of FMT on disease symptoms or characteristics were rare. In addition, several neurological disorders tend to fluctuate in disease severity, which implicates that assessment of FMT efficacy might be difficult. The mechanism of action of FMT might be different in different disorders: while in its consolidated use for recurrent *C. difficile* infections usually a single FMT is sufficient to produce sustained benefit, for neurological disorders, which are progressive in nature, multiple FMT may be necessary to achieve a sustained response; the required number of FMTs and effective interval between multiple FMTs would then need to be evaluated. For a few case descriptions and studies on ASD and PD, indeed only transient positive effects were observed, but this may also be explained by a placebo effect or fluctuations in disease severity.

For some neurological disorders examined in this review, contrasting evidence was observed in gut microbiota analysis results. Various non-standardized methods could be used for the assessment of the gut microbiota composition. The results of microbiota analyses can be affected by a wide variety of factors throughout the entire workflow of a microbiota study, starting with sample collection and DNA extraction and ending with choice of statistical tests. In addition, due to the influence of many other patient- or subject related factors, including medication use, diet, or age, inconsistency in gut microbiota composition and α- and β-diversity analyses is often observed. Furthermore, functional analyses of the gut microbiome, using metagenomics and metabolomics, are emerging and may be more important than the detection of taxa, exclusively reported by many studies. What the gut bacteria produce and the concomitant effects on processes in the human body is more important information than which bacteria are present.

The safety of this experimental treatment should also be better elucidated. Potential benefits of FMT should be carefully weighed against the potential risks, and future studies should focus primarily on safety, with effectivity of FMT as a secondary endpoint. Indeed, not all human and animal studies mentioned assessment of adverse events in subjects undergoing FMT and potential long-term negative effects were rarely examined. Side effects could be related to the administration route (e.g., gastroscopy or colonoscopy) (Wang et al., 2016), to the required pretreatment (antibiotics and bowel lavage), or even to the administration of feces itself, which could theoretically influence the pathogenesis of the disorder in a negative way. Transferring certain taxa or inducing an increase in α-diversity may turn out to be beneficial or detrimental, and this may differ between individual disorders. Whether all healthy donors or only a few donors with certain gut microbiome characteristics are suitable, is unknown. Rational feces donor selection, based on available literature, may be crucial, but more knowledge on the pathophysiology of several neurological disorders and the relevant characteristics of the microbiome is required. Also, future studies should answer the question whether gut microbiota baseline profiles can predict who will benefit from FMT. Herein, the use of appropriate methods for the (preparation of) microbiota analysis and assessment of possible confounding factors are crucial. Confounding factors should be corrected for or prevented. Concomitantly, functional analyses of the gut microbiome will have a crucial role in determining which taxa are important in each neurological disorder. Also important is that more evidence has to become available on the number of FMTs required for each neurological disorder. Furthermore, it is unclear whether pre-treatment with antibiotics and bowel lavage is necessary in neurological disorders. The current view in *C. difficile* infections is that pre-treatment improves donor feces engraftment. In our opinion, a similar pre-treatment as in *C. difficile* infections may also be required to foster the effects of FMT in neurological disorders. In the future, capsules that contain beneficial bacterial consortia may replace FMT, thus increasing the comfort for patients and reducing the potential side effects associated with the administration route.

In conclusion, although some evidence is available, well-designed large double-blinded randomized controlled trials in human patients are needed to further elucidate the effect of FMT in neurological disorders.

## Author Contributions

KV reviewed all literature and wrote the manuscript. PJ assembled the figure, performed the initial search, and participated in writing the manuscript. RO, JL, and BO reviewed the literature on MS and wrote the section of the manuscript on MS. EK and MC supported and supervised the writing of the manuscript. QD aided in the interpretation of microbiota data. All authors critically reviewed the manuscript.

### Conflict of Interest

KV, RO, JK, and EK are members of the Netherlands Donor Feces Bank (https://www.ndfb.nl/), which received an unrestricted grant from Vedanta Biosciences in Boston (https://www.vedantabio.com/). The remaining authors declare that the research was conducted in the absence of any commercial or financial relationships that could be construed as a potential conflict of interest.

## Abbreviations

3AIBA: 3-aminoisobutyric acid
3-CST: three-chamber sociability test
5-HT: serotonin or 5-hydroxytryptamine
A: aged 18–20 months old mice
AB: antibiotics
ABC: aberrant behavior checklist
AC: amoxicillin/clavulanate
AC Res: amoxicilline/clavulanate-resistant
AC Sens: amoxicilline/clavulanate-sensitive
AD: Alzheimer's disease
AE: adverse event
Ampho-B: amphotericin B
APP: Aβ precursor protein
APPPS1: co-expression of KM670/671NL Swedish mutation of human amyloid precursor protein (APP) and L166P mutation of human presenilin 1 (PS1) under control of the Thy-1 promoter with age-dependent Aβ parenchymal accumulation and minimal vascular Aβ amyloid, restricted to the pial vessels
APPPS1-21: amyloid precursor proteinSWE/ presenilin 1L166P mouse model of amyloid-β amyloidosis/Alzheimer's disease that express familial AD–linked APPSWE and PS1L166P transgenes driven by the neuron-specific Thy1 promoter
ASD: autism spectrum disorder
ASO: alpha-synuclein overexpression
Aβ: amyloid beta
cAPPPS1: APPPS1 mice with conventional microbiota
CARS: childhood autism rating scale
CD: cognitive dysfunction
CDi: control diet
CD11b: a type of mouse antigen-specific antibodies
CDAI: Crohn's disease activity index
CFA: complete Freud's adjuvant
CLDN1: claudin 1
cMCAO: permanent occlusion of MCA distal of lenticulostriate arteries (cause small cortical lesions)
CNS: central nervous system
Conv-11168: mice are infected with C. jejuni from enteric disease patient
Conv-260.94: mice are infected with C. jejuni from GBS patient
Conv-TSB: mice are inoculated with tryptic soy broth
d: day(s)
DA: striatal dopamine
DSI: direct social interaction test
DSR: daily stool records
EAE: experimental autoimmune encephalomyelitis
EDSS: expanded disability status scale
fMCAO: transient occlusion of MCA by temporarily placing a filament in the internal carotid artery
FMD: fasting mimicking diet
FMT: fecal microbiota transplantation
FPD: Faith's phylogenetic diversity
FST: forced swimming test
GABA: gamma-aminobutyric acid
GBS: Guillain-Barré syndrome
GF: germ-free
GI: gastrointestinal
GSI: gastrointestinal severity index
GSRS: gastrointestinal symptom rating scale
HC: healthy control
HHC: human healthy household control
HK: heat-killed
HN: high intensity noise
Hu: humanized
HWT: hang wire test
i.c.: ileocecocolic
i.p.: intraperitoneal
IL: interleukin
KCNA1: Potassium Voltage-Gated Channel Subfamily A Member 1
KDi: ketogenic diet
LN: low-intensity noise
LPS: lipopolysaccharide
m: month(s)
MB: marble burying test
MCAO: middle cerebral artery occlusion
mNSS: modified neurological severity score
MOG: myelin oligodendrocyte glycoprotein
MPTP: 1-methyl-4-phenyl-1,2,3,6-tetrahydropyridine
(SP)MS: (secondary progressive) multiple sclerosis
MSFC: Modified Multiple Sclerosis Functional Composite
MWMT: morris water maze test
MWT: mechanical withdrawal test
N: normal
NA: data not available
ND: normally developing
NDS: neurological deficit score
Non-anh: spared nerve injury without developing a anhedonia-like phenotype
NF: mice that were treated with normal saline by intraperitoneal injection and fasting mimicking diet
non-sign.: non-significant
NS: normal saline
OFT: open-field testing
OLT: object location test
ORT: novel object recognition test
OTU: operational taxonomic unit
PAC-QOL: patient assessment of constipation–quality of life
PANDAS: pediatric autoimmune neuropsychiatric disorders associated with streptococcal infections syndrome
PANS: pediatric acute-onset neuropsychiatric syndrome
PBS: phosphate-buffered solution
PBS/G: mice that were treated with 20% glycerol in sterile phosphate-buffered solution
PCoA: principal coordinates analysis
PD: Parkinson's disease
PGI-III: parent global impressions-III
PGI-R: parent global impressions-revised
phylog. div: phylogenetic diversity
PLS-DA: partial least squares discrimination analysis
PPA: propionic acid
Qol: quality of life
RR: relapsing-remitting
SAE: serious adverse event(s)
SAMP8: senescence-accelerated mouse prone 8
SAMR-1: senescence-accelerated mouse resistant 1
SC: sodium citrate buffer (control for FMT)
SCFA: short chain fatty acids
SD: Sprague Dawley
SDI: stroke dysbiosis index
SDI-H: stroke patients with a high stroke dysbiosis index
SDI-L: stroke patients with a low stroke dysbiosis index
SGHM: Standardized Human Gut Microbiota
SNI: spared nerve injury
SPF: specific-pathogen-free
SPT: Sucrose preference test
SRS: Social Responsiveness Scale
STER: mice are handled sterile
SW: Swiss-Webster
T1D: type 1 diabetes mellitus
TET: Transendoscopic enteral tubing
TFT: Tail-flick test
Th: T-helper cells, Thy1-αSyn alpha-synuclein-overexpression mouse model
TLR: toll-like receptor
TNF: tumor necrosis factor
Treg: regulatory T cells
TS: Tourette syndrome
TSB: tryptic soy broth
TST: tail suspension test
UPDRS: unified Parkinson's disease rating scale
USV: ultrasonic vocalizations test
VABS-II: Vineland Adaptive Behavior Scale II
VAS: Visual analog scale
w: week(s)
w.: weighted
WT: wild-type
y: year(s)
y.o.: year old
Y: young 8-12 weeks old mice
YGTSS: Yale Global Tic Severity Scale
ZO-1: tight junction protein 1.
