# Supplementary material for: Fecal Microbiota Transplantation in Neurological Disorders
Source: Front Cell Infect Microbiol. 2020 Mar 24;10:98. doi: 10.3389/fcimb.2020.00098 (PMC7105733; doi:10.3389/fcimb.2020.00098)
Supplement: Supplementary file 1 [file Data_Sheet_1.pdf]

Supplementary Material

1 Appendix 1: Search strategy

On the 23rd of July 2019, a search of the literature about FMT in neurological diseases was performed in Pubmed, Embase, Web of Science, COCHRANE library and Academic Search Premier database. Articles or meeting abstracts were included. Furthermore, the reference lists of some recent reviews were consulted.

The following search strategy was used:

((("Fecal Microbiota Transplantation"[mesh] OR fecal transplant*[tw] OR faecal transplant*[tw] OR feces transplant*[tw] OR fecal microbiota transplant*[tw] OR faecal microbiota transplant*[tw] OR "Fecal Microbiota Transplantation"[tw] OR "Faecal Microbiota Transplantation"[tw] OR "Fecal Microbiota Transplantations"[tw] OR "Fecal Transplantation"[tw] OR "Fecal Transplantations"[tw] OR "Faecal Transplantation"[tw] OR "Faecal Transplantations"[tw] OR "Fecal Transplant"[tw] OR "Fecal Transplants"[tw] OR "Faecal Transplant"[tw] OR "Faecal Transplants"[tw] OR "Feces Transplantation"[tw] OR stool transplant*[tw] OR "stool transplant"[tw] OR "stool transplantation"[tw] OR "fecal bacteriotherapy"[tw] OR "faecal bacteriotherapy"[tw] OR microbiota transplant*[tw] OR microbiota-transplant*[tw] OR "microbiota transfer"[tw] OR "microbiota-transfer"[tw] OR "fecal transfer"[tw] OR "faecal transfer"[tw] OR (("Feces"[mesh] OR "fecal"[tw] OR "faecal"[tw] OR "feces"[tw] OR "faeces"[tw]) AND ("FMT"[tw] OR "Transplantation"[mesh:noexp] OR "transplantation"[Subheading] OR "transplantation"[tw] OR transplant*[tw]))) AND ("Nervous System Diseases"[mesh] OR "Hepatic Encephalopathy"[mesh] OR "Hepatic encephalopathy"[tw] OR "Neuromyelitis Optica"[mesh] OR "neuromyelitis optica"[tw] OR "Alzheimer Disease"[mesh] OR Alzheimer*[tw] OR "Huntington Disease"[mesh] OR Huntington*[tw] OR "Amyotrophic Lateral Sclerosis"[mesh] OR "amyotrophic lateral sclerosis"[tw] OR "Autism Spectrum Disorder"[mesh] OR "Autism"[tw] OR asperger*[tw] OR "autistic"[tw] OR "Diabetic Neuropathies"[mesh] OR neuropath*[tw] OR "Dementia"[mesh] OR "dementia"[tw] OR neurodegenerat*[tw] OR neurologic disease*[tw] OR neurologic disorder*[tw] OR "nervous system disease"[tw] OR "nervous system diseases"[tw] OR "Peripheral Nervous System Diseases"[mesh] OR "sarcopenia"[tw] OR "sarcopenic"[tw] OR "sarcopenia"[mesh] OR epilep*[tw] OR "epilepsy"[mesh] OR "stroke"[tw] OR "stroke"[mesh] OR "cerebral infarction"[tw] OR "cerebral infarction"[mesh] OR "cerebral ischemia"[tw] OR "cerebrovascular ischemia"[tw] OR "brain infarction"[tw] OR "brain infarction"[mesh] OR "brain ischemia"[tw] OR "brain ischemia"[mesh] OR "rett syndrome"[tw] OR "rett syndrome"[mesh] OR "Guillain barre"[tw] OR "Guillain-barre"[tw] OR "Guillain barré"[tw] OR "Guillain-barré"[tw] OR "Guillain-barre syndrome"[mesh] OR "migraine"[tw] OR "migraine disorders"[mesh] OR "Myalgic Encephalomyelitis"[tw] OR "Encephalomyelitis"[mesh] OR "multiple system atrophy"[tw] OR "multiple system atrophy"[mesh] OR "Parkinsonian Disorders"[Mesh] OR "Parkinson disease"[tw] OR "Parkinson's disease"[tw] OR "Parkinsons disease"[tw] OR "Parkinson"[tw] OR parkinson*[tw] OR "Lewy Body Disease"[tw] OR "Lewy Body"[tw] OR "MPTP Poisoning"[tw] OR "multiple sclerosis"[tw] OR "multiple sclerosis"[mesh] OR "experimental autoimmune encephalitis"[tw] OR "experimental autoimmune encephalitis"[tw] OR "tourette"[tw] OR tourette*[tw] OR "Tourette syndrome" [mesh] OR "tic disorder"[tw]) AND (english[la] OR dutch[la])) OR (("Gut Microbiota"[tw] OR "Gastrointestinal

Microbiome"[mesh] OR "gut microbiome"[tw] OR "gut microbiomes"[tw] OR "gastrointestinal microbiome"[tw] OR "gastrointestinal microbiomes"[tw] OR "gut micro biota"[tw] OR "gut micro ecology"[tw] OR "gut micro environment"[tw] OR "gut micro flora"[tw] OR "gut micro organisms"[tw] OR "gut microbe"[tw] OR "gut microbes"[tw] OR "gut microbial"[tw] OR "gut microbiology"[tw] OR "gut microbioma"[tw] OR "gut microbiota"[tw] OR "gut microbiotas"[tw] OR "gut microecology"[tw] OR "gut microenvironment"[tw] OR "gut microenvironments"[tw] OR "gut microflora"[tw] OR "gut microorganism"[tw] OR "gut microorganisms"[tw] OR "Gut Flora"[tw] OR "gut microbial communities"[tw] OR "gut microbial community"[tw] OR "gastrointestinal micro organisms"[tw] OR "gastrointestinal microbe"[tw] OR "gastrointestinal microbes"[tw] OR "gastrointestinal microbiology"[tw] OR "gastrointestinal microbiota"[tw] OR "gastrointestinal microbiotas"[tw] OR "gastrointestinal microecology"[tw] OR "gastrointestinal microenvironment"[tw] OR "gastrointestinal microflora"[tw] OR "gastrointestinal microorganisms"[tw] OR "Gastrointestinal Flora"[tw] OR "gastrointestinal microbial communities"[tw] OR "gastrointestinal microbial community"[tw] OR "intestinal microbiome"[tw] OR "intestinal microbiomes"[tw] OR "intestinal micro ecology"[tw] OR "intestinal micro environment"[tw] OR "intestinal micro flora"[tw] OR "intestinal micro organisms"[tw] OR "intestinal microbe"[tw] OR "intestinal microbes"[tw] OR "intestinal microbial"[tw] OR "intestinal microbiology"[tw] OR "intestinal microbiota"[tw] OR "intestinal microbiotas"[tw] OR "intestinal microecology"[tw] OR "intestinal microenvironment"[tw] OR "intestinal microenvironments"[tw] OR "intestinal microflora"[tw] OR "intestinal microorganism"[tw] OR "intestinal microorganisms"[tw] OR "Intestinal Flora"[tw] OR "intestinal microbial communities"[tw] OR "intestinal microbial community"[tw] OR "intestine microbiome"[tw] OR "intestine microbe"[tw] OR "intestine microbial"[tw] OR "intestine microbiota"[tw] OR "intestine microecology"[tw] OR "intestine microflora"[tw] OR "intestine microorganism"[tw] OR "intestine microorganisms"[tw] OR "enteric microbiome"[tw] OR "enteric micro environment"[tw] OR "enteric micro organisms"[tw] OR "enteric microbes"[tw] OR "enteric microbiology"[tw] OR "enteric microbiota"[tw] OR "enteric microenvironment"[tw] OR "enteric microflora"[tw] OR "enteric microorganism"[tw] OR "enteric microorganisms"[tw] OR "Enteric Flora"[tw] OR "enteric microbial communities"[tw] OR "enteric microbial community"[tw] OR "gut bacteria"[tw] OR "gastrointestinal bacteria"[tw] OR "intestinal bacteria"[tw] OR "enteric bacteria"[tw] OR "colonic microbiome"[tw] OR "colonic micro flora"[tw] OR "colonic micro organisms"[tw] OR "colonic microbes"[tw] OR "colonic microbial"[tw] OR "colonic microbiota"[tw] OR "colonic microenvironment"[tw] OR "colonic microflora"[tw] OR "colonic microorganisms"[tw] OR "Colonic Flora"[tw] OR "colonic microbial community"[tw] OR "colonic microbiome"[tw] OR "colonic micro flora"[tw] OR "colonic micro organisms"[tw] OR "colonic microbes"[tw] OR "colonic microbial"[tw] OR "colonic microbiota"[tw] OR "colonic microenvironment"[tw] OR "colonic microflora"[tw] OR "colonic microorganisms"[tw] OR "Colonic Flora"[tw] OR "colonic microbial community"[tw] OR "colon microbiome"[tw] OR "colon microbes"[tw] OR "colon microbiology"[tw] OR "colon microbiota"[tw] OR "colon microenvironment"[tw] OR "colon microflora"[tw] OR "Colon Flora"[tw] OR "colon microbial community"[tw] OR "duodenal microbiome"[tw] OR "duodenal micro environment"[tw] OR "duodenal microbiota"[tw] OR "duodenal microflora"[tw] OR "Duodenal Flora"[tw]) AND ("Transplantation"[mesh:noexp] OR "transplantation"[Subheading] OR "transplantation"[tw] OR transplant*[tw]) AND ("Nervous System Diseases"[mesh] OR "Hepatic Encephalopathy"[mesh] OR "Hepatic encephalopathy"[tw] OR "Neuromyelitis Optica"[mesh] OR "neuromyelitis optica"[tw] OR "Alzheimer Disease"[mesh] OR Alzheimer*[tw] OR "Huntington Disease"[mesh] OR

Huntington*[tw] OR "Amyotrophic Lateral Sclerosis"[mesh] OR "amyotrophic lateral sclerosis"[tw] OR "Autism Spectrum Disorder"[mesh] OR "Autism"[tw] OR asperger*[tw] OR "autistic"[tw] OR "Diabetic Neuropathies"[mesh] OR neuropath*[tw] OR "Dementia"[mesh] OR "dementia"[tw] OR neurodegenerat*[tw] OR neurologic disease*[tw] OR neurologic disorder*[tw] OR "nervous system disease"[tw] OR "nervous system diseases"[tw] OR "Peripheral Nervous System Diseases"[mesh] OR "sarcopenia"[tw] OR "sarcopenic"[tw] OR "sarcopenia"[mesh] OR epilep*[tw] OR "epilepsy"[mesh] OR "stroke"[tw] OR "stroke"[mesh] OR "cerebral infarction"[tw] OR "cerebral infarction"[mesh] OR "cerebral ischemia"[tw] OR "cerebrovascular ischemia"[tw] OR "brain infarction"[tw] OR "brain infarction"[mesh] OR "brain ischemia"[tw] OR "brain ischemia"[mesh] OR "rett syndrome"[tw] OR "rett syndrome"[mesh] OR "Guillain barre"[tw] OR "Guillain-barre"[tw] OR "Guillain barré"[tw] OR "Guillain-barré"[tw] OR "Guillain-barre syndrome"[mesh] OR "migraine"[tw] OR "migraine disorders"[mesh] OR "Myalgic Encephalomyelitis"[tw] OR "Encephalomyelitis"[mesh] OR "multiple system atrophy"[tw] OR "multiple system atrophy"[mesh] OR "Parkinsonian Disorders"[mesh] OR "Parkinson disease"[tw] OR "Parkinson's disease"[tw] OR "Parkinsons disease"[tw] OR "Parkinson"[tw] OR parkinson*[tw] OR "Lewy Body Disease"[tw] OR "Lewy Body"[tw] OR "MPTP Poisoning"[tw] OR "multiple sclerosis"[tw] OR "multiple sclerosis"[mesh] OR "experimental autoimmune encephalitis"[tw] OR "experimental autoimmune encephalitis"[tw] OR "tourette"[tw] OR tourette*[tw] OR "Tourette syndrome" [mesh] OR "tic disorder"[tw]) AND (english[la] OR dutch[la]))**)**

In addition, ClinicalTrials.gov was searched on the 27th of June 2019 for planned or ongoing clinical trials with keywords fecal microbiota transplantation and by the topic of Nervous System Diseases.

2 Appendix 2: Planned and ongoing studies

NCT nr	Title	Conditions	Interventions	Status	Characteristics	Sponsors/collaborators
NCT03998423	Oral fecal microbiota transplant feasibility study in Alzheimer's disease	Alzheimer's disease	Biological: Fecal microbiota transplantation	Recruiting	Study Type: Interventional<br>Phase: Phase 1<br>Allocation: Randomized<br>Intervention Model: Parallel Assignment<br>Masking: Open Label	University of Wisconsin, Wisconsin partnership program
NCT03766321	Fecal microbiota transplantation effect on amytrophic lateral sclerosis patients	Amytrophic lateral Sclerosis	Biological: FMT, placebo	Not yet recruiting	Interventional, phase not applicable, RCT. Parralel assignment, quadrupel marked	Azienda spedaliero universitaria di Modena, University of modena and Reggio Emelia, Catholic university of the sacred heart, Campus bio-medico university, Azienda Ospedaliero universitaria careggi, Azienda ospedliera di Perugia, University of Chieti, University of Florence
NCT03829878	Efficacy, safety, and tolerabitility study of oral full-spectrum microbiota TM (CP101) in subjects with autism spectrum disorder and associated GI symptoms (SPROUT)	Autism spectrum disorder, autism, ASD, autistic thinking, autistic behavior, finch, FMT, fecal microbiota transplantation, CP101, SPROUT, fecal transplant	Drug: CP101 & placebo	Not yet recruiting	Interventional phase 2 placebo-controlled RCT. Quadrupel masking, crossover assignment	Finch Research and development LLC
NCT03426826	The gut-brain study	Autism spectrum disorder, gastro-intestinal disorder	Biological: FMT vs Placebo	Recruiting	Interventional, phase 1 RCT, parallel assignment, quadrupel masked	Children's Hospital Los Angeles
NCT03408886	Microbiota transfer therapy for adults with autism spectrum disorder who have GI disorders	Autism spectrum disorder, GI disorder	Drug: vancomycin, MoviPrep, Biological: Full spectrum microbiota	Recruiting	Interventional, phase 2 RCT, parallel assignment, quadrupel masked	Arizona state University

NCT03183889	FMT in relapsing MS patients	Autoimmune diseases, relapsing MS	Drug: FMT	Recruiting	Interventional, phase 2 RCT, crossover assignment, no masking	Lawson Health Research institute
NCT03594487	FMT of FMP30 in relapsing-remitting multiple sclerosis	Relapsing remitting multiple sclerosis	Drug: FMP30 donor stool, Procedure: FMT of FMP30 donor stool,	Recruiting	Interventional, phase 1, non randomized, parallel assignment, no masking	University of California, San FranciscoA
NCT03975413	Fecal microbiota transplantation (FMT) in multiple sclerosis	Multiple sclerosis, relapsing-remitting	Fecal microbiota transplantation (FMT)	Active, not recruiting	Prospective case-only observational study	Rush University Medical Center
NCT02889627	FMT for epilepsy	Epilepsy	Drug: Fecal microbiota suspension	Recruiting	Interventional phase 2 & 3, single group assignment, no masking	The second hospital of nanjing medical university
NCT03876327	Fecal microbiota transplantation as a potential treatment for Parkinson's disease	Parkinson's disease	Procedure: Fecal microbiota transplantation	Recruiting	Interventional, open labelled, phase 2 & phase 3 non-randomized trial	Soroka University Medical center
NCT03808389	Fecal microbiota transplantation for Parkinson's disease	Parkinson's disease	Other: donor FMT & autologous FMT	Recruiting	Interventional RCT, quadrupel masked.	University of Ghent, The Flanders institute for Biotechnology, Reserch foundation Flanders, Parkinson's disease patient organizations: Vlaamse Parkinson Liga, Parkili
NCT03671785	Study of the fecal microbiome in patients with Parkinson's disease	Parkinson's disease	Drug: PRIM-DJ727, placebo	Not yet recruiting	Interventional, phase 1 RCT, parallel assignment, single masked	The university of Texas Health science center houston, Kelsey Research Foundation

3 Appendix 3: Explanation of abbreviations and terms

24:A-FMT: 24-weeks old mice receiving FMT with feces from aged mice

24:Y-FMT: 24-weeks old mice receiving FMT with feces from young mice

3AIBA: 3-aminoisobutyric acid

3-CST: three-chamber sociability test that assesses general sociability and interest in social novelty

5-HT: serotonin or 5-hydroxytryptamine

A: aged 18-20 months old mice

A+A-FMT: FMT in aged mice with feces from aged mice

A+Y-FMT: FMT in aged mice with feces from young mice

AB (+/-) MCAO+SPF-FMT: mice that received 8 weeks of AB (stopped 48 hours before FMT) and FMT with feces from specific-pathogen-free mice and subsequently induction of MCAO 4 days after FMT

AB (+/-) sham: mice that undergo sham-surgery with 8 weeks of antibiotic treatment stopped 72 h before surgery

AB (+/-) sham: SPF-FMT mice that received 8 weeks of AB (stopped 48 hours before FMT) and FMT with feces from specific-pathogen-free mice and subsequently sham-surgery 4 days after FMT

AB (+/+) sham: mice that undergo sham-surgery with antibiotic treatment during the entire experiment

AB (+/-) MCAO: mice with induction of MCAO with 8 weeks of antibiotic treatment stopped 72 h before surgery

AB (+/+) MCAO: mice with induction of MCAO with antibiotic treatment during the entire experiment

AB: antibiotics

AB+MPTP+NF/HK: mice were treated with AB and MPTP intraperitoneal injection and heat-killed (HK) gut microbiota by gastric gavage from mice that were treated with normal saline by intraperitoneal injection and fasting mimicking diet

AB+MPTP+PBS/G: mice were treated with AB and MPTP intraperitoneal injection and 20% glycerol in sterile phosphate-buffered solution by gastric gavage

ABC: test that assesses problem behaviors in five areas common in children with ASD (irritability, lethargy, stereotypy, hyperactivity, inappropriate speech) called Aberrant Behavior Checklist

AC: amoxicillin/clavulanate

AC Res: amoxicilline/clavulanate-resistant

AC Res FMT+MCAO: treated with FMT with feces from AC-resistant mice before MCAO

AC Sens: amoxicilline/clavulanate-sensitive

AC Sens FMT+MCAO: treated with FMT with feces from AC-sensitive mice before MCAO

AD: Alzheimer’s disease

AE: adverse event

AL: ad libitum diet

Ampho-B: amphotericin B

Anh: anhedonia-like phenotype

Anh-FMT: FMT in mice with feces from rats that underwent spared nerve injury and that developed a anhedonia-like phenotype

APP: Aβ precursor protein

APPPS1: co-expression of KM670/671NL Swedish mutation of human amyloid precursor protein (APP) and L166P mutation of human presenilin 1 (PS1) under control of the Thy-1 promoter with age-dependent Aβ parenchymal accumulation and minimal vascular Aβ amyloid, restricted to the pial vessels

APPPS1-21: amyloid precursor proteinSWE/ presenilin 1L166P mouse model of amyloid-β amyloidosis/Alzheimer’s disease that express familial AD–linked APPSWE and PS1L166P transgenes driven by the neuron-specific Thy1 promoter

APPPS1-21-FMT: Mice that were treated with high-dose AB and thereafter received FMT with feces from age-matched APPPS1-21 male donor mice (recipients had low-dose AB in the water during entire experiment, donor received no AB)

ASD: autism spectrum disorder

ASO: alpha-synuclein overexpression

Aβ: amyloid beta

cAPPPS1: APPPS1 mice with conventional microbiota

cAPPPS1-FMT: 4 months old GF APPPS1 mice that received FMT with feces from aged (12 months) old APPPS1 mice with conventional microbiota

CARS: Test for diagnosing autism and ASD and assessment of overall severity of symptoms called Childhood Autism Rating Scale

CD: cognitive dysfunction

CDi: control diet

CD11b: a type of mouse antigen-specific antibodies

CDAI: Crohn's disease activity index

CFA: complete Freud's adjuvant

Chao1: estimates microbiota diversity from abundance data (measure of richness)

CLDN1: claudin 1

cMCAO: permanent occlusion of MCA distal of lenticulostriate arteries (cause small cortical lesions)

CNS: central nervous system

Conv-11168: mice are infected with C. jejuni from enteric disease patient

Conv-260.94: mice are infected with C. jejuni from GBS patient

Conv-TSB: mice are inoculated with tryptic soy broth

cWT-FMT: 4 months old GF APPPS1 mice that received FMT with feces from aged (12 months) old WT mice with conventional microbiota

cylinder test: test to assess animal's overall activity by examining forepaw use and asymmetry

d: day(s)

DA: striatal dopamine

DSI: direct social interaction test which is a test on interaction with a stimulus mouse, that is recorded using an overhead video camera

DSR: rating of stool using the Bristol Stool Form scale called daily stool records

EAE: experimental autoimmune encephalomyelitis

EDSS: expanded disability status scale

fMCAO: transient occlusion of MCA by temporarily placing a filament in the internal carotid artery

fMCAO+WT-FMT: fMCAO was performed and on the same day FMT with feces from WT mice

fMCAO-FMT+cMCAO: FMT with feces from mice that underwent fMCAO and after 3 days cMCAO was performed

FMD: fasting mimicking diet, fasting 3 days followed by 4 days of refeeding for three 1-week cycles

FMT: fecal microbiota transplantation

Foxp3 expression: a marker for neuroprotective Treg cells

FPD: Faith's phylogenetic diversity

FST: forced swimming test, in which mice are placed individually in a cylinder with water. The immobility time for mouse is recorded for 5 min.

GABA: gamma-aminobutyric acid

GBS: Guillain-Barré syndrome

GF: germ-free

GF+human HC-FMT: GF mice that received FMT with feces from human healthy controls

GF+human PD-FMT: GF mice that received FMT with feces from human PD patients

GF+SPF-WT-FMT: GF mice that received FMT with feces from SPF WT mice

GF-KDi: GF mice that received a ketogenic diet

GF-KDi+SPF-FMT: GF mice that received a ketogenic diet after they have received FMT with feces from SPF mice

GI: gastrointestinal

GSI: gastrointestinal severity index

GSRS: assessment of GI symptoms during the previous week scored in five domains (abdominal pain, reflux, indigestion, diarrhea, constipation) called Gastrointestinal Symptom Rating Scale

HC: healthy control

HHC: human healthy household control

HHC-FMT: FMT with feces from a human healthy household control

HK: heat-killed

HN: high intensity noise

HT: healthy twin

HT-FMT: mice received FMT with feces from human healthy twin (MS-discordant monozygotic twins)

Hu: humanized

Hu-11168: mice are infected with C. jejuni from enteric disease patient and are offspring of mice receiving FMT with feces from human healthy controls

Hu-260.94: mice are infected with C. jejuni from GBS patient and are offspring of mice receiving FMT with feces from human healthy controls

human-AD-FMT: mice received FMT with feces from Alzheimer’s disease patients

human-HC-FMT: mice received FMT with feces from human healthy controls

Hu-TSB: mice are inoculated with tryptic soy broth and are offspring of mice receiving FMT with feces from human healthy controls

HWT: a test to assess motor strength called hang wire test

i.c.: ileocecocolic

i.p.: intraperitoneal

IL: interleukin

KCNA1: Potassium Voltage-Gated Channel Subfamily A Member 1

KDi: ketogenic diet

LN: low-intensity noise

LPS: lipopolysaccharide

m: month(s)

MB: marble burying test

MB: marble burying test, which is a test on anxiety or obsessive–compulsive disorder behavior based on the observation that mice will bury harmful or harmless objects in their bedding

MCA: middle cerebral artery

MCAO control: mice that underwent MCAO induction and no antibiotic treatment

MCAO: middle cerebral artery occlusion

mNSS: modified neurological severity score

MOG: myelin oligodendrocyte glycoprotein

MPTP: 1-methyl-4-phenyl-1,2,3,6-tetrahydropyridine

MPTP+AL-FMT: mice received MPTP and FMT with feces from mice that were treated with normal saline by intraperitoneal injection and were fed ad libitum

MPTP+FMD-FMT: mice that received MPTP and FMT with feces from mice that were treated with normal saline by intraperitoneal injection and fasting mimicking diet

MPTP+HC-FMT: mice that received an MPTP injection i.p. and then a FMT with feces from normal control WT mice

MPTP+PBS: mice that received MPTP i.p. and then PBS by gavage

(SP)MS: (secondary progressive) multiple sclerosis

MSFC: Modified Multiple Sclerosis Functional Composite

MS-FMT: mice received FMT with feces from MS-human donors

MWMT: test for memory and spatial learning called Morris water maze test

MWT: mechanical withdraw test, in which mice receive electronic filaments to the lateral 1/3 of their right paws. The quick withdrawal of their paws or flinching is considered a positive response.

N: normal

NA: data not available

ND: normally developing

NDS: neurological deficit score

N-FMT: FMT with feces from normal hamsters

Non-anh: spared nerve injury without developing a anhedonia-like phenotype

Non-anh-FMT: FMT in mice with feces from rats that underwent spared nerve injury without developing a anhedonia-like phenotype

NF: mice that were treated with normal saline by intraperitoneal injection and fasting mimicking diet

non-sign.: non-significant

NS: normal saline

NS+HC-FMT: mice that received NS intraperitoneally and then a FMT with feces from normal control WT mice

NS+PBS: mice that received NS intraperitoneally and then PBS by gavage

NS+PD-FMT: mice that received NS intraperitoneally and then a FMT with feces from MPTP-mice

OFT: test used to explore general locomotor activity levels, anxiety, and willingness to explore called open-field testing

OLT: object location test which assesses spatial memory and discrimination

ORT: novel object recognition test which assesses recognition memory

OTU: operational taxonomic unit

PAC-QOL: patient assessment of constipation–quality of life

PANDAS: pediatric autoimmune neuropsychiatric disorders associated with streptococcal infections syndrome

PANS: pediatric acute-onset neuropsychiatric syndrome

PBS: phosphate-buffered solution

PBS/G: mice that were treated with 20% glycerol in sterile phosphate-buffered solution

PCoA: principal coordinates analysis

PD: Parkinson’s disease

Peyer’s patches: part of gut associated lymphoid tissue

PGI-III: questionnaire for parents about observed behavioural changes called parent global impressions-III

PGI-R: parent global impressions-revised

phylog. div: phylogenetic diversity

PLS-DA: partial least squares discrimination analysis

PPA: propionic acid

probe trial: a test to evaluate spatial memory. A platform below the water surface in a water pool was removed from the pool and mice were allowed to swim freely for 60 s in any quadrant. The number of platform crossings and time spent in each quadrant were recorded

Qol: quality of life

Rag1-/-: lymphocyte deficient mice

RR: relapsing-remitting

SAE: serious adverse event(s)

SAMP8: senescence-accelerated mouse prone 8

SAMP8-FMT: mice receiving feces from control senescence-accelerated mouse prone 8 mice

SAMP8-HN-FMT: mice receiving feces from senescence-accelerated mouse prone 8 mice, that were subject to high intensity noise for 30 days

SAMR-1: senescence-accelerated mouse resistant 1

SAMR1-FMT: mice receiving feces from senescence-accelerated mouse resistant 1 mice

SC: sodium citrate buffer (control for FMT)

SCFA: short chain fatty acids

SD: Sprague Dawley

SDI: stroke dysbiosis index

SDI-H: stroke patients with a high stroke dysbiosis index

SDI-H-FMT: mice received FMT with feces from stroke patients with a high stroke dysbiosis index

SDI-L: stroke patients with a low stroke dysbiosis index

SDI-L-FMT: mice received FMT with feces from stroke patients with a low stroke dysbiosis index

SGHM: Standardized Human Gut Microbiota

sham: animals that underwent a sham-intervention

Sham-FMT: FMT with feces from animals that had undergone a sham-intervention

Sham-FMT+cMCAO: FMT with feces from mice that received sham-surgery and after 3 days cMCAO was performed

Sham-stress: Sprague Dawley rats that were placed in plastic restraining cones for 5 min

Shannon: Shannon diversity index which accounts for both abundance and evenness of species

Short-AB (+/-) MCAO: mice with antibiotic treatment for 48 h up to 72 h before MCAO-surgery

Simpson: Simpson's diversity index which accounts for both abundance and evenness of species

SNI: spared nerve injury

SPF: specific-pathogen-free

SPF+AB: SPF mice that received antibiotics

SPF-CDi: SPF mice that received a control diet

SPF-CDi+CDi-FMT: SPF mice that received a control diet after they have received FMT with feces from SPF mice receiving a control diet

SPF-CDi+KDi-FMT: SPF mice that received a control diet after they have received FMT with feces from SPF mice receiving a ketogenic diet

SPF-KDi: SPF mice that received a ketogenic diet

SPF-KDi+CDi-FMT: SPF mice that receive a ketogenic diet after they have received FMT with feces from SPF mice receiving a control diet

"SPT: Sucrose preference test, in which animals are exposed to water and a 1% sucrose solution for 48 hours, followed by 24 hours of water and food deprivation with 24 hours exposure to two identical bottles, of which one is water and the other is the sucrose solution. The bottles contain-

ing water and sucrose are weighed and the preference of sucrose is measured."

SRS: test with 65-item scale that assesses social impairments called Social Responsiveness Scale

STER: mice are handled sterile

stress: Sprague Dawley rats that were placed in plastic restraining cones for 2 h to induce chronic stress

stress-FMT: Sprague Dawley rats that received FMT with feces from stressed rats

SW: Swiss-Webster

T1D: type 1 diabetes mellitus

T1D-CD-FMT: Mice receiving FMT with feces from type 1 diabetes mellitus mice that have cognitive dysfunction

T1D-Non-CD-FMT: Mice receiving FMT with feces from type 1 diabetes mellitus mice that have no cognitive dysfunction

TET: Transendoscopic enteral tubing

TFT: Tail-flick test in which the latency time to withdrawal or flick of the tail of mice that have their tail in hot water is measured

Th: T-helper cells

Thy1-αSyn: alpha-synuclein-overexpression mouse model

TLR: toll-like receptor

TNF: tumor necrosis factor

Treg: regulatory T cells

TS: Tourette syndrome

TSB: tryptic soy broth

"TST: tail suspension test, in which a small piece of adhesive tape is placed 2 cm from the tip of the tail of mice. The immobility

time is recorded by camera, which is defined as the time hung passively and completely motionless."

unw.: unweighted

UPDRS: unified Parkinson's disease rating scale, a test used to follow the longitudinal course of PD, containing motor- and non-motor assessments evaluated by interview and clinical observation.

USV: test on social interactions that evaluates communication with specialized sounds in the ultrasonic range called ultrasonic vocalizations

VABS-II: measure of the functioning level in four different domains (communication, daily living skills, motor skills and socialization) and 11 sub-domains called Vineland Adaptive Behavior Scale II

VAS: Visual analogue scale

vehicle: control group for FMT, mice received another solution instead of a fecal suspension, administered in the same manner as the fecal suspension in the FMT-group with the same pre-treatment

w: week(s)

w.: weighted

WT: wild-type

y: year(s)

y.o.: year old

Y: young 8-12 weeks old mice

Y+A-FMT: FMT in young mice with feces from aged mice

Y+Y-FMT: FMT in young mice with feces from young mice

YGTSS: Yale Global Tic Severity Scale

ZO-1: tight junction protein 1
